# Internet of Things-Cloud Control of a Robotic Cell Based on Inverse Kinematics, Hardware-in-the-Loop, Digital Twin, and Industry 4.0/5.0

**DOI:** 10.3390/s25061821

**Published:** 2025-03-14

**Authors:** Dan Ionescu, Adrian Filipescu, Georgian Simion, Adriana Filipescu

**Affiliations:** 1 Department of Automation, “Dunarea de Jos” University of Galati, 800008 Galati, Romania; dan.ionescu@ugal.ro (D.I.); georgian.simion@ugal.ro (G.S.); 2 Doctoral School of Fundamental Sciences and Engineering, “Dunarea de Jos” University of Galati, 800008 Galati, Romania

**Keywords:** VC, DT, A/RC, Industry 4.0/5.0, Digital Twin, HIL, PLC, SCADA, HMI, OPC-UA, STPN

## Abstract

The main task of the research involves creating a Digital Twin (DT) application serving as a framework for Virtual Commissioning (VC) with Supervisory Control and Data Acquisition (SCADA) and Cloud storage solutions. An Internet of Things (IoT) integrated automation system with Virtual Private Network (VPN) remote control for assembly and disassembly robotic cell (A/DRC) equipped with a six-Degree of Freedom (6-DOF) ABB 120 industrial robotic manipulator (IRM) is presented in this paper. A three-dimensional (3D) virtual model is developed using Siemens NX Mechatronics Concept Designer (MCD), while the Programmable Logic Controller (PLC) is programmed in the Siemens Totally Integrated Automation (TIA) Portal. A Hardware-in-the-Loop (HIL) simulation strategy is primarily used. This concept is implemented and executed as part of a VC approach, where the designed PLC programs are integrated and tested against the physical controller. Closed loop control and RM inverse kinematics model are validated and tested in PLC, following HIL strategy by integrating Industry 4.0/5.0 concepts. A SCADA application is also deployed, serving as a DT operator panel for process monitoring and simulation. Cloud data collection, analysis, supervising, and synchronizing DT tasks are also integrated and explored. Additionally, it provides communication interfaces via PROFINET IO to SCADA and Human Machine Interface (HMI), and through Open Platform Communication—Unified Architecture (OPC-UA) for Siemens NX-MCD with DT virtual model. Virtual A/DRC simulations are performed using the Synchronized Timed Petri Nets (STPN) model for control strategy validation based on task planning integration and synchronization with other IoT devices. The objective is to obtain a clear and understandable representation layout of the A/DRC and to validate the DT model by comparing process dynamics and robot motion kinematics between physical and virtual replicas. Thus, following the results of the current research work, integrating digital technologies in manufacturing, like VC, IoT, and Cloud, is useful for validating and optimizing manufacturing processes, error detection, and reducing the risks before the actual physical system is built or deployed.

## 1. Introduction

In the manufacturing and automation industry, adopting new Industry 4.0/5.0 concepts like advanced manufacturing technology and intelligent digital technology integration has become essential for achieving success in a fast-evolving and competitive market. Industry 4.0/5.0 plays a key role in engineering digital transformation by introducing smart manufacturing technologies like IoT and digitalization into manufacturing and industrial processes.

Digital Twin technology involves a virtual transformation of the field, by shifting and replacing traditional, physical factories to a more digital-focused approach model. It achieves this by incorporating and integrating advanced software tools and technologies to enhance business factory operations and processes. As part of the DT technology, the VC technique is used, employing a virtual environment process, for simulating and testing the system off-site, typically in the context of manufacturing or industrial automation.

Digital transformation and smart factories are fundamental concepts in modern-day industrial manufacturing and automation, defined by the integration of digital technologies such as the IoT, DT, VC, Cloud robotics, and storage in big data analytics [1]. DT is a dynamic, clear, and understandable representation, a virtual replica of the physical system that offers a detailed, real-time perspective on asset information, mirroring system behavior, properties, and interactions. In the manufacturing industry, a DT is a virtual model and process visualization platform that integrates and accurately replicates the characteristics, performance, and operations of the actual physical system [2,3].

The integration of twins into engineering processes is rapidly accelerating, primarily driven by Industry 4.0/5.0. This advancement brings “smart” manufacturing system capabilities into the digital realm, enhancing connectivity and facilitating improvements across performance indicators, production, system support, prediction, efficiency, and quality and reducing operational costs and risks [4,5,6].

As part of the DT technology, the VC tool and techniques are used to increase production and performance on a long-term basis by optimizing the operations tasks, to improve the traceability by monitoring, analyzing, and tracking every aspect of all manufacturing processes like costs, risks, and reducing delays associated with the real on-site commissioning. It is well known that a variety of potential problems can arise when the manufacturing automation system is first deployed, during integration with IO Field and actual controller (PLC): software bugs or obsolete/unnecessary functions, machine sequence and operations faults, sensors, or communication issues. Therefore, DT is used to perform commissioning for equipment and systems virtually first, to minimize the downtime risks and errors during real, on-site commissioning. The main goal is to identify potential problems at an early stage that can be solved at the virtual environment level before the real process is put into operation.

**Figure 1:**
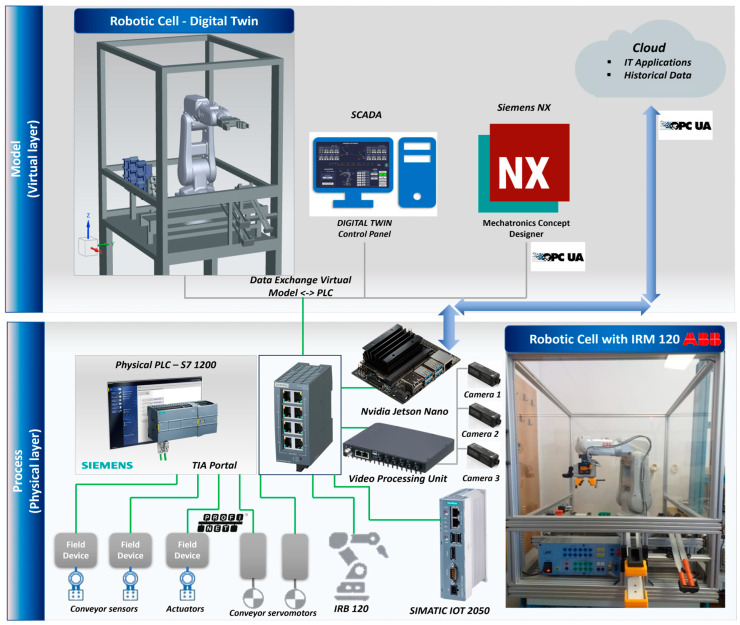
A/DRC with ABB 120 IRM. DT Physical and Virtual Layer Communication topology.

In this paper, we focus mainly on the design, modeling, and software implementation of the DT robotic cell platform application, as seen in Figure 1, by developing a virtual replica for an A/DRC assisted by ABB 120 IRM for assembly and disassembly of a predefined five-component product [7], evaluating through extensive testing its accuracy, reliability, effectiveness, and applicability in real-world applications. The primary motivation for developing and testing a DT robotic cell platform application is to ensure that the virtual design and behavior accurately reflect the dynamics of the physical system. This is achieved by validating the 3D model implementation and the analytical methods of the IRM inverse kinematics model. The objective is to implement a complex virtual environment development using cutting-edge digital technology for remote monitoring, analyzing, designing, and optimizing engineering processes and operations without requiring direct intervention on the physical equipment. Consequently, the presented manufacturing technology facilitates the integration of the VC concept by leveraging the bi-directional data exchange flow between the dynamic digital representation of the physical system. By allowing the integration of IoT into Cloud infrastructure, a stable, flexible tech backbone is built, this digital platform provides a safe and robust tool for real-time remote monitoring with optimized performance, simulating scenarios, and predicting maintenance needs or potential outcomes, ultimately leading to smarter decision making and improved overall efficiency [8].

The PLC software design introduces a new approach, beyond the logic for IRM and A/DRC automation control, referred to as the physical layer. It includes a set of PLC function blocks for kinematic computation, translating models from MATLAB R2021a to PLC code for use in the digital simulation environment of the complete engineering process. Additionally, it provides communication interfaces via PROFINET IO to SCADA and HMI, and through OPC UA for Siemens NX MCD with a DT virtual model. A closed-form inverse kinematics solution for the ABB 120 IRM is implemented in the PLC, using the geometric approach, which generates and selects the right solution according to robot configuration input in a reasonably faster time due to the analytic nature of the computation method.

The primary contribution aspects and originality of this research refer especially to the software development of an HIL simulator to emulate a robotic cell which allows a real-time interface with its physical counterpart through advanced analytics. Our primary focus was on software implementation and optimization [9], IoT-Cloud Control and data acquisition, as well as 3D virtual modeling methods of A/DRC physical process dynamics, followed by simulation using MATLAB and ABB RobotStudio. Additionally, we integrated the bi-directional data exchange flow between the digital model and the physical process. In this research, the DT approach for mirroring a flexible robotic cell is an extremely powerful tool for predictive maintenance and optimization to improve performance. It can be used additionally as a design and planning tool for logging, reporting, and control. We discuss several system elements that we designed in the laboratory:

- Siemens 1200 PLC program for controlling ABB 120 IRM and mobile visual servoing to be able to perform movements, positioning, and storage of disassembled parts;
- Development of a Siemens NX 3D virtual model as a dynamic replica of the DT’s physical counterpart;
- Virtual model control, based on inverse kinematics computation, has been designed to facilitate a digital simulation environment for the entire physical process, from modeling in MATLAB to PLC code implementation;
- Additionally, path planning for linear motion, using the MoveL command from ABB(ABB Ltd., Zurich, Switzerland), from an initial to a desired final position is implemented in the PLC, utilizing a smooth Cubic Polynomials function;
- Implementation and optimization of Siemens 1200 PLC software to execute emulated robotic cell assembly and disassembly functions for HIL-structured DT design;
- Development of an IoT cloud-based DT for real-time sensor data logging and reporting in MongoDB 8.0;
- OPC UA interface connectivity, virtual and real mapping, real-time interaction with the ‘real-world’ between DT and A/DRC related parameters;
- SCADA interactive graphic interface programming in Siemens TIA Portal;
- Interactive graphic interface programming of HMI KTP700.

Simulation results will be compared with real-world experimental data to evaluate the DT’s predictability, reliability, and overall performance. Moreover, this study explores the complex communication and control of A/DRC, assessing the integration and compatibility of IoT and Cloud-based infrastructures. It also addresses DT scalability and adaptability, ensuring its applicability to larger industrial systems and multiple applications.

The rest of the paper is organized as follows: hardware structure, basic design concept, and architecture of the A/DRC assisted by ABB 120 IRM, IoT integrated within the DT application, and the Cloud environment, is presented in Section 2. The theoretical framework of 6-DOF RM kinematics is addressed in Section 3, highlighting the analytical methods for both the calculation and implementation of inverse kinematics. Robotic cell task operations and modeling using Petri Nets are also discussed. This section covers 3D virtual model implementation as well, using Siemens NX MCD software. Section 4 focuses on the design, implementation, and testing of a DT application including PLC program design. In this section, VC-“smart” manufacturing systems as new concepts of Industry 4.0/5.0 are introduced and the integration with DT for manufacturing systems is discussed. SCADA serves as a DT operator control interface for collecting, monitoring, and visualizing live data or Cloud history within the framework of the presented DT technology is described in Section 5. In this section, the integration of remote solutions via Cloud or VPN is also presented, exploring Node-RED application for building powerful, smart IoT integrated automation system. A/DRC testing and simulations for virtual model operations, as well as real-time control results for DT and Cloud solution implementation, are essential key points and will be discussed in Section 6. Section 7 is focused on discussing the outcomes, analyzing data, and reporting findings. The last section, Section 8, outlines the conclusions of the proposed approach.

## 2. A/DRC System Characterization

### 2.1. Hardware Structure of the A/DRC

Regarding flexible manufacturing technology within an A/DRC environment, a system has been designed to allow the complete product assembly and disassembly process on the station assisted by ABB 120 IRM [10]. The entire process was designed to meet the requirements for adapting the initial manufacturing system to a flexible one, allowing A/D tasks for two technical different workpieces, in the same cell.

The robotic cell, shown in Figure 1, comprises a Siemens S7-1200 PLC-controlled A/D unit, equipped with SCADA supervision with IO field data collection and monitoring capabilities. This station manages various tasks at the operational level, from storing and supplying subcomponents to delivering the fully assembled product onto the cell’s conveyor unit, which functions as a transport line. Although the presented robotic cell is a laboratory setup, it employs the ABB 120 IRM to recreate, mirror, and simulate a real industrial process by executing multiple A/D tasks [11]; the last, is to place the final built product (assembly) or to recover disassembled components and place them into the respective storage units on the side of the cell station (disassembly). Each final product comprises five components for assembly: base pallet, body, top, and two inserted cylinders, which can be either plastic or metal [12]. The assembly process is carried out through IRM repetitive operations of picking up and handling/transporting components to the designated assembly area within the cell. Thus, the system is equipped with storage areas for the components needed for assembly; ABB 120 IRM picks up the necessary components and completes each assembly operation in full. In the case of the disassembly task, the reverse assembly process occurs, PLC is handling operations while the disassembled components are picked up and placed by RM to their corresponding storage areas from the robotic cell. The complete system setup is built on a centralized PLC architecture, synchronized with the DT environment, to operate the ABB 120 IRM equipped with an electric gripper, enabling coordinated management, motion planning, and control of all subsystems. A/DRC control strategy employs IRM feedback of joint angles to the S7-1200 PLC, receives signals from the sensors located on the conveyor, processes camera images, and transmits control signals to the actuating elements, as well as interfaces the controller of the conveyor motor.

As mentioned, the basic design concept of the hardware architecture integrates three major subsystems, which operate, communicate, and synchronize together: PLC, SCADA, and DT 3D-virtualization of the complete process using Siemens NX MCD [13], using PROFINET and OPC UA interfaces, acting as a complete flexible manufacturing cell with DT functionality that performs synchronized tasks for operations and functionality of the engineering process.

PROFINET interface enables HMI and SCADA to communicate with PLC, providing greater interoperability [14]. Therefore, several assembly tasks can be assigned to the A/DRC station, followed by the disassembly operation of the subcomponents using ABB 120 IRM. Process monitoring sensors are connected to the digital inputs of the PLC, components such as solenoid valves and actuators are connected to the PLC’s digital outputs while Jetson Nano using Node-RED [15], helps integration of cell’s sensors to IoT application within DT and Cloud platform using OPC UA. Complex execution components, such as servomotor drivers, are also connected and controlled by the PLC via the PROFINET protocol. Thus, this system can be classified as an IoT system from Industry 4.0/5.0 perspectives. This type of communication enables quick and easy information data exchange between the robotic cell system devices, sensors, and the transport system, enabling communication technologies and applications over the Internet.

An HMI linked to the system’s network facilitates the interconnection between the manufacturing system and the external decision-making environments. This allows the operator to quickly and efficiently input the necessary production data setpoints—such as the number of products and the configuration of the intermediate layer of parts for each product. The HMI also enables the operator to control and monitor the production process, providing access to information regarding the status of robotic cell workstations and production progress. Moreover, the HMI connection to the system’s PROFINET network enables fast and precise communication with all other cell workstations and integrated devices. This ensures swift communication, as well as good synchronization between workstations, thus, avoiding potential issues and errors that may arise during production.

To identify the type and position of components, A/DRC uses a visual identification system in the control loop, with integrated video cameras into the IRM workspace, a system called visual system (VS) [12,16,17]. Data captured by the visual system is processed within the robot controller, to pick up the necessary components and to position them in the desired locations. Following the processed information from the VS 3D HD cameras, and from the robotic cell IO Field Devices, the control, positioning, and tracking tasks are synchronized and executed by the ABB 120 IRM using PLC commands [12].

### 2.2. ABB 120 IRM

ABB 120 IRM is used in this research. Designed for industrial environments, robots feature specific types of capabilities, like advanced motion control technology with an open-source Robot automation Framework including Rapid programming language, which simplifies complex motion paths and operations providing communication and easy integration in a variety of industrial automation system setups [18]. The six-axis configuration gives it high flexibility, with each axis offering a specific range of motion and a great Smooth Path Control for precise path following. This configuration creates a comprehensive 3D workspace. Full rotational reach and extended flexibility in end-effector orientation provides usability in compact environments, particularly when mounted on various surfaces. The robot is controlled with the IRC5 ABB compact controller and control software, RobotWare 6.15.07 build 7032, which supports every aspect of the robot system, such as motion control, system integration, and communication with third-party devices, enabling simulation and offline programming development, which minimizes setup time and optimizes performance [19]. ABB 120 IRM performances and product detailed specifications per axes are shown in Table 1.

The end-effector robot arm position data and target to where is heading are communicated by robot IRC5 controller at any cycle time to PLC using a *robtarget* data type format. *Robtarget* data type consists of four components:

- Translation—position ${(P}_{x}{,P}_{y}{,P}_{z})\;$ of the end effector expressed in mm;
- Rotation—orientation, expressed in the form of quaternions ${(q}_{1}{,q}_{2}{,q}_{3}{,q}_{4})$;
- Robot configuration—axis configuration of the robot ${(\mathrm{cf}}_{1}{,\mathrm{cf}}_{4}{,\mathrm{cf}}_{6}{,\mathrm{cf}}_{x})$. The configuration supervision will check axes 1, 4, and 6, will not move more than 180 degrees;
- External axes—position of the external axes (eax_a,eax_b,eax_c,eax_d,eax_e,eax_f). The robot controls up to six external axes, in addition to its six internal axes, each logical axis can be connected to a physical axis, in this case, the connection must be activated in the system parameters.

Rotation quaternions or Euler parameters arranged in quaternion form, as seen in ABB *robtarget* position data type, describe the axis–angle representation, a commonly used method for defining any 3D rotation (or sequence of rotations). This representation can serve as an alternative to rotation matrices in the three-dimensional space, by specifying only two parameters: a unit vector for the rotation axis and the rotation angle about an axis. Therefore, for a given unit vector axis (x,y,z), to which a rotation angle, ω, is applied, the Euler parameters can be defined using the following conversion:

(1) $$q_{1}=\cos\frac{\omega }{2};\;q_{2}=x\text{·}\sin\frac{\omega }{2};{\;q}_{3}=y\text{·}\sin\frac{\omega }{2};{\;q}_{4}=z\text{·}\sin\frac{\omega }{2}$$

Given the rotation quaternion for the end effector orientation, as defined in ABB:

(2) $$q=(q_{1},{\;\;q}_{2},{\;\;q}_{3},{\;\;q}_{4})\;$$

(3) $$q_{1}=\omega ;{\;q}_{2}={\;P}_{x};\;q_{3}={\;P}_{y};{\;q}_{4}={\;P}_{z},$$

The corresponding transformation matrix, T, for robot direct kinematics, can be calculated in terms of the unit quaternion components:

(4) $$\left[\begin{array}{cccc} \omega^{2}+x^{2}-y^{2}-z^{2} & \;2\text{·}(xy\;-\;\omega z) & \;2\text{·}(\omega y\;+\;xz) & P_{x}\\ 2\text{·}(\omega z\;+\;xy) & \omega^{2}-x^{2}+y^{2}-z^{2} & 2\text{·}(yz\;-\;\omega x) & P_{y}\\ 2\text{·}(xz\;-\;\omega y) & 2\text{·}(\omega x\;+\;yz) & \;\omega^{2}-x^{2}-y^{2}+z^{2} & P_{z}\\ 0 & 0 & 0 & 1 \end{array}\right]$$

When calculating the corresponding axis positions (joint angles) through inverse kinematics, for the six-axis robot, there will be eight possible solutions generated [20,21,22]. This means that the robot can reach the same end effector position and orientation, with several different link angles or configurations for the RM axes [23]. For reducing/selecting the desired joint angles solution ${(\theta }_{1},\theta_{2},\theta_{3}{,\;\theta }_{4},\theta_{5},\theta_{6})$ out of the eight possible, the RM controller provides a subset of data through the *robtarget* data type; therefore, the robot configuration data type is specified using axis 1, 4, and 6 quadrant values. For a rotating axis, the configuration values define the quadrant of the robot axis meaning a 90° quarter revolution in a positive or negative direction. Component ${\mathrm{cf}}_{x}$ is used to select one of eight possible robot configurations numbered from 0 through 7. When the RM movement is finalized, the RM controller checks that the robot has reached the programmed configuration.

## 3. Modeling and Simulation of the A/DRC

### 3.1. ABB 120 IRM Kinematics. Modeling, Analysis, and Validation

#### 3.1.1. Forward Kinematics

The direct kinematics general approach is based on linear algebra, to compute the joint variables function of the RM mechanical structure for the initial, reference frame [23].

For kinematic chain representation in a more compact way, the homogeneous transformation matrix is preferred to represent a frame, translated to a new position P and rotated over another frame, described by the fundamental rotational matrices [24], as follows:

(5) Ann−1=R(θn)nn−1P(θn)nn−101X3σ,

where σ is the scale factor, which usually is 1.

For the forward kinematics approach and calculation of the 6-DoF RM position and orientation, the homogenous transformation matrix A, representing how a frame Σ must be displaced to achieve the next frame, will be renamed as T matrix, to describe further the complete kinematic chain by using post-multiplication proprieties. The Denavit–Hartenberg (D-H) convention is used [25,26] to describe the open kinematic chain structure, which introduces four parameters (scalars). These parameters vary with the RM joint motion, to represent a coordinate frame, Σ, displacement in space to achieve the next one.

The graphical representation and interpretation of the DH parameters for describing each robot link are as follows:

- $\theta_{i}$—angle/ rotation between axis $X_{i-1}\;$ and axis $X_{i}$, about the axis $Z_{i-1}$, which varies with the joint motion;
- $d_{i}$—coordinate of the origin **O_i_** translation distance along the $Z_{i-1}$ axis of the previous frame;
- $\alpha_{i}$—the rotation angle between axis $Z_{i-1}\;$ and axis $Z_{i}$ about the axis $X_{i}$;
- $a_{i}$—the translation distance between origin coordinates $O_{i}$ and $O_{i-1}$, along axis $X_{i}$.

**Figure 2:**
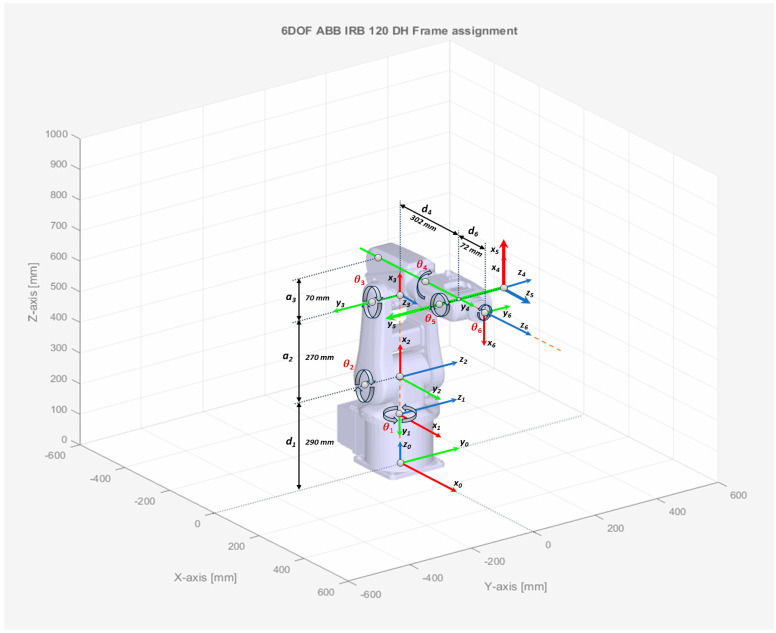
D-H link coordinate system and frame assignment for the ABB 120 IRM.

According to the dimensions provided by ABB in the product specifications for ABB 120 IRM, D-H kinematic parameters are shown in Table 2, while the D-H link coordinate system and frame assignment for the robot joints are presented further in Figure 2. To represent the relation between two frames Σ and $\Sigma_{i-1}$ such as the representation or specification of the orthogonal coordinate frames, by translating or rotating the frame $\Sigma_{i-1}\;$ to achieve the next frame $\Sigma_{i}$, the joint-to-joint homogenous transformation matrix $T_{i}$ is used, where i>1. For every joint, i, greater than 1, associated with the degree of freedom, each transformation matrix is a function of $\theta_{i}$ Angle, which gives the relationship between the frame $\Sigma_{i}$ over the frame $\Sigma_{i-1}$, starting with the initial frame, $\Sigma_{0}$. Therefore, using the D-H parameters, for every link or joint coordinate i, representing each of the 6 links, such that 1<i≤m where m is 6, $T_{i}\;$ can be written, by successive post multiplication of the single transformations [27].

**Figure 3:**
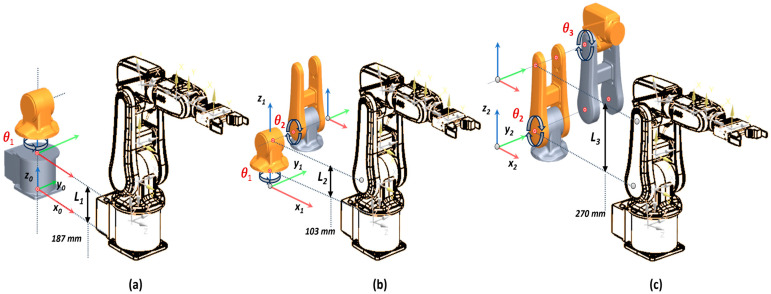
ABB 120 IRM frame transformations according to D-H convention: (**a**) frame Σ_0_ to frame Σ_1_; (**b**) frame Σ_1_ to frame Σ_2_; (**c**) frame Σ_2_ to frame Σ_3_.

The D-H parameters from Table 2, defined by the manipulator kinematic parameters, are substituted into the homogeneous transformation matrices, sometimes called relative transforms, for generating the joint-to-joint transformation matrices for each frame and link dimension [24], which maps the coordinates of adjacent links i−1 over the link *i*. The initial frame, $\Sigma_{0}$, is moved to achieve the next frame $\Sigma_{1}$, origin $O_{1}$, and rotated with $\alpha_{1}$ about the *x*-axis. The translation distance along the *z*-axis is $L_{1}$, as pointed in Figure 3a.

(6) T(θ1)10=Rz(θ1)10P(θ1)1001X31=cos(θ1)−sin(θ1)00sin(θ1)cos(θ1)00001L10001

Frame $\Sigma_{2}$ is achieved by shifting $\Sigma_{1}$ with $L_{2}$ along the *z*-axis and rotated with $\theta_{2}$ about the *z*-axis (Figure 3b).

(7) T(θ2)21=Ry(θ2)21P(θ2)2101X31=cos(θ2)0sin(θ2)00100−sin(θ2)0cos(θ2)L20001

Frame $\Sigma_{3}$ is achieved by shifting $\Sigma_{2}$ with $L_{3}$ along the *z*-axis and rotated with $\alpha_{3}$ about the *x*-axis (Figure 3c).

(8) T(θ3)32=Ry(θ3)32P(θ3)3201X31=cos(θ3)0sin(θ3)00100−sin(θ3)0cos(θ3)L30001

**Figure 4:**
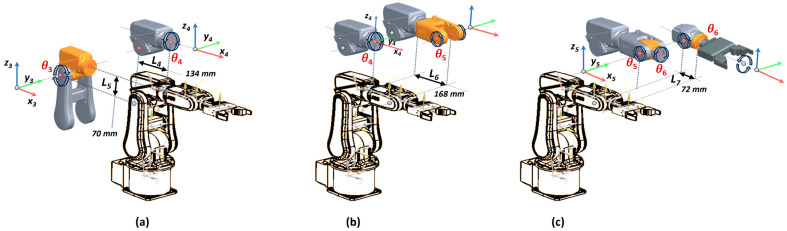
ABB 120 IRM frame transformations according to D-H convention: (**a**) frame Σ_3_ to frame Σ_4_; (**b**) frame Σ_4_ to frame Σ_5_; (**c**) frame Σ_5_ to frame Σ_6_.

Frame $\Sigma_{4}$ is achieved by shifting $\Sigma_{3}$ with $L_{4}$ along the *x*-axis, shifting with $L_{5}$ along the *z*-axis and rotated with $\alpha_{4}\;$ about the *x*-axis (Figure 4a).

(9) T(θ4)43=Rx(θ4)43P(θ4)4301X31=100L40cos(θ4)−sin(θ4)00sin(θ4)cos(θ4)L50001 

Frame $\Sigma_{5}$ is achieved by shifting $\Sigma_{4}$ with $L_{6}$ along the *x*-axis and rotated with $\alpha_{5}$ about the *x*-axis (Figure 4b).

(10) T(θ5)54=Ry(θ5)54P(θ5)5401X31=cos(θ5)0sin(θ5)L60100−sin(θ5)0cos(θ5)00001

Frame $\Sigma_{6}$ is achieved by shifting $\Sigma_{5}$ with $L_{7}$ along the *x*-axis and rotated with $\theta_{6}$ about the *z*-axis (Figure 4c).

(11) T(θ6)65=Rx(θ6)65P(θ6)6501X31=100L70cos(θ6)−sin(θ6)00sin(θ6)cos(θ6)00001 

Matrix Tii−1 denotes the homogeneous transformation, by relating the description of a point in the frame $\Sigma_{i}$ to the description of the same point in the frame $\Sigma_{i-1}$. The algebraic approach of forward kinematics can be expressed as a matrix successive concatenation, by multiplying all six transformation matrices according to the D-H convention [27,28,29]. This allows the complete transformation from the origin of the base robot to the RM end effector by splitting the transformation matrix T60 into matrices, which generate a rotation matrix and a translation vector to calculate the position and orientation of a kinematic chain from the joint variables.

(12) T60=T · 10T · 21T · 32T · 43T · 54T65=nxsxaxPxnysyayPynzszazPz0001

By extracting the fourth column of T, the end effector position vector p is achieved. Furthermore, n is the normal vector, s the sliding vector, and a is the approach vector:

(13) $$p=\left[\begin{array}{c} P_{x}\\ P_{y}\\ P_{z} \end{array}\right],\;\;n=\left[\begin{array}{c} n_{x}\\ n_{y}\\ n_{z} \end{array}\right],\;\;s=\left[\begin{array}{c} s_{x}\\ s_{y}\\ s_{z} \end{array}\right],\;\;a=\left[\begin{array}{c} a_{x}\\ a_{y}\\ a_{z} \end{array}\right]$$

By defining the D-H parameter table and calculating the homogeneous transformation matrix, T, a mathematical representation of the robot forward kinematic structure is achieved. To calculate the inverse kinematics solutions, the homogeneous transformation matrix, T, is the starting point, referred to as the input for the inverse kinematics problem. In this scenario, for reaching a desired position and orientation of the end-effector, each joint coordinates are calculated, associated with the degree of freedom denoted by the set of unknown variables ${(\theta }_{1},\theta_{2},\theta_{3}{,\;\theta }_{4},\theta_{5},\theta_{6})$.

#### 3.1.2. Inverse Kinematics

There are several ways to calculate the inverse kinematics solution, which can be tested concerning each other, the geometric approach—used further in this paper, as well as the algebraic method which can be solved by equation systems using numerical methods [21]. The analytical solution derives a closed-form mathematical expression to directly compute joint angles or positions based on the desired end-effector position and orientation providing explicit, precise expressions for joint configurations without approximation, enabling rapid computation, unlike numeric solutions. The motion algorithms embedded in most industrial robot controllers avoid implementing numerical solutions of Jacobian Inverse Kinematics Algorithms. Instead, they employ analytical inverse solutions, which are computationally more efficient.

**Figure 5:**
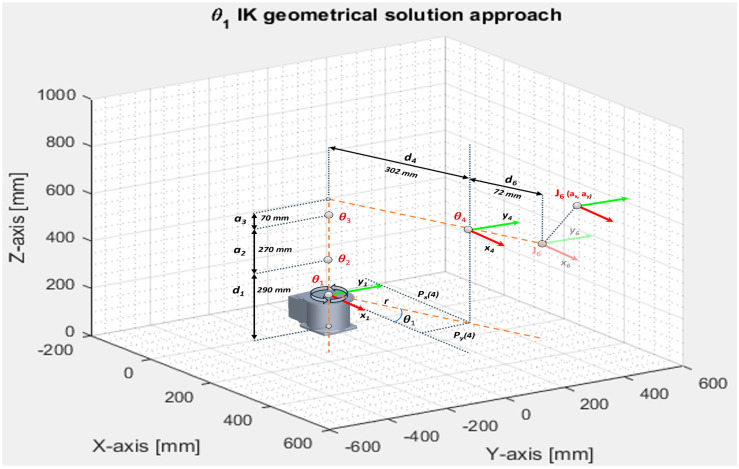
Joint $\theta_{1}$ geometrical solution approach by computing and reducing into an XY-plane the position of the wrist, $P_{4}$.

To simplify the problem, the kinematic decoupling method is implemented by splitting the inverse kinematics general problem into two separate directions using the relative position and orientation of the intersection of the three reference systems in one point: first homogenous matrix T30 defines the position and second matrix T63 for solving the orientation problem of the spherical wrist. The geometric solution for the first joint angle is determined by reducing the three-dimensional problem into an XY-plane, taking into consideration only the first three joints and keeping in mind that the wrist motion of the fourth coordinate system is not influencing the equation. The first angle, $\theta_{1}$, is calculated by computing the position of the wrist, $P_{4}$, from the rotation matrix, T, by using the approach vector, a, and position vector, p, of the end effector position (Figure 5). The geometric solution for the inverse kinematics of the first link angle, $\theta_{1}$, admits two possible solutions, although it is not always physically or mechanically possible. $\theta_{1}$ and ${(\theta }_{1}+\pi )$ are calculated as below:

(14) $$P_{4}=p-d_{6}\cdot a,\;$$

(15) $$p=\left[\begin{array}{c} P_{x}\\ P_{y} \end{array}\right],\;\;a=\left[\begin{array}{c} a_{x}\\ a_{y} \end{array}\right]$$

(16) $$\theta_{1}=atan2(P_{4}\left(y\right),\;{\;P}_{4}\left(x\right))\;$$

(17) $$\gamma =arccos\frac{{a_{2}}^{2}+R^{2}-{L_{4}}^{2}}{2R\cdot a_{2}}$$

(18) $$\;\;\beta =atan2(P_{4}\left(z\right)-d_{1},r)\;\;$$

(19) $$\eta =arccos\frac{{a_{2}}^{2}+{L_{4}}^{2}-R^{2}}{2a_{2}\cdot L_{4}}$$

(20) $$\varphi =atan2(d_{4},a_{3})$$

(21) $$L_{4}=\sqrt[{\;}]{{a_{3}}^{2}+{d_{4}}^{2}}$$

(22) $$r=\sqrt{{P_{4}\left(x\right)}^{2}+{P_{4}\left(y\right)}^{2}}$$

(23) $$R=\sqrt{r^{2}+({P_{4}\left(z\right)-d_{1})}^{2}}\;$$

**Figure 6:**
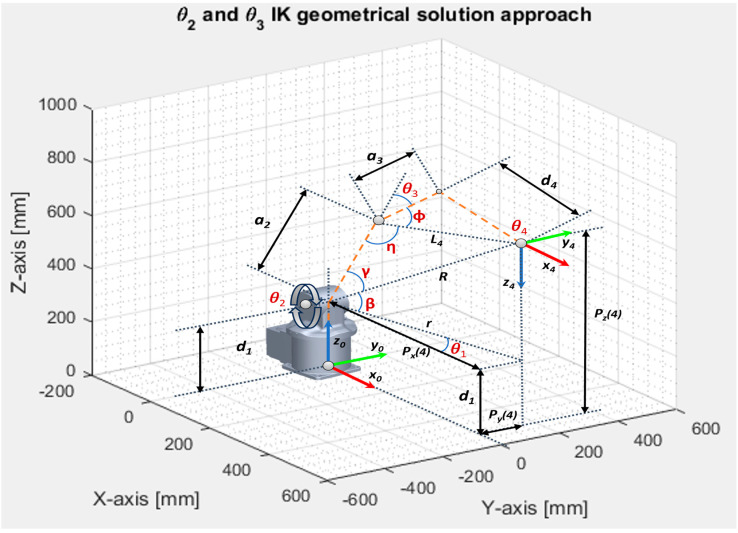
Joints $\theta_{2}$ and $\theta_{3}$ geometrical solution approach starting from the computation of the wrist position $P_{4}$ and joint angle $\theta_{1}$.

Geometric solution for the inverse kinematics of the joint angle $\theta_{2}$ and $\theta_{3}$, as drafted in Figure 6, admits two solutions.

For elbow up:

(24) $$\theta_{2}=\frac{\pi }{2}-\beta -\gamma \;$$

(25) $$\theta_{3}=\pi -\varphi -\eta \;$$

For elbow down:

(26) $$\theta_{2}=\frac{\pi }{2}-\beta +\gamma$$

(27) $$\theta_{3}=\pi -\varphi +\eta \;\;$$

As discussed, by applying the kinematic decoupling approach and using the desired orientation of the end effector, the last three joint angles can be calculated by finding a set of Euler angles corresponding to the desired rotation matrix, R63, which is equal to the rotation matrix for the Euler angles [28,29].

(28) T=R 30· R63 

(29) R63=(R63)−1 · T=(R32)−1 · (R21)−1 · (R10)−1 · T

(30) R63=r11r12r13r21r22r23r31r32r33=c4·c5·c6−s4·s6−c4·c5·s6−s4·c6c4·s5s4·c5·c6+c4·s6−s4·c5·s6+c4·c6s4·s5−s5·c6s5·c6c5 

For the nonsingular case, meaning $\theta_{5}\neq 0$, which implies ${cos(\theta }_{5})\neq 1$, there are two possible geometric solutions for the inverse kinematics orientation:

(31) $$r_{33}=cos\left(\theta_{5}\right)\;\;$$

(32) $$sin\left(\theta_{5}\right)=\pm \sqrt{1-{\left(cos\left(\theta_{5}\right)\right)}^{2}}=\pm \sqrt{1-{r_{33}}^{2}}\;$$

(33) $$\theta_{5}=atan2(r_{33},\pm \sqrt{1-{r_{33}}^{2}})$$

For wrist up:

(34) $$\theta_{4}=atan2(r_{23},r_{13})$$

(35) $$\theta_{6}=atan2(-r_{32},r_{31})$$

For wrist down:

(36) $$\theta_{4}=atan2(r_{23},r_{13})\;+\pi$$

(37) $$\theta_{6}=atan2(-r_{32},r_{31})+\pi$$

For the singular configuration $\theta_{5}=0$, meaning ${cos(\theta }_{5})$ in the matrix R63 is 1, it generates an infinite number of solutions (degenerate solution, in this case, $\theta_{4}\;$ cannot be determined), only the sum of $\left(\theta_{4}+\theta_{6}\right)$ can be found; thus, it is common to arbitrarily set $\theta_{4}$ to null and solve the $\theta_{6}$ joint angle.

(38) $$\theta_{4}+\theta_{6}=atan2(r_{21},r_{11})\;\;$$

(39) R63=c4 · c6−s4 · s6−c4 · s6−s4 · c60s4 · c6+c4 · s6−s4 · s6+c4 · c60001

For wrist up:

(40) $$\;\;\theta_{4}=0\;\;$$

(41) $$\theta_{6}=atan2(r_{12}-r_{21},{-r}_{11}-r_{22})$$

For wrist down:

(42) $$\theta_{4}=-\pi$$

(43) $$\theta_{6}=atan2(r_{12}-r_{21},{-r}_{11}-r_{22})+\pi$$

**Figure 7:**
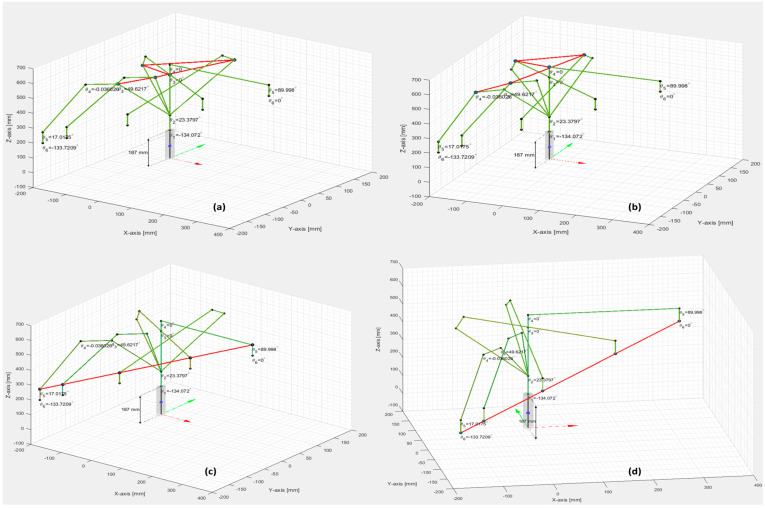
ABB 120 IRM Linear motion space simulation with Inverse Kinematics: (**a**) $\theta_{3}\;$ joint angle; (**b**) $\theta_{4}\;$ joint angle; (**c**) $\theta_{5}\;$ joint angle; (**d**) $\theta_{6}\;$ joint angle.

Figure 7 presents the simulation results of the inverse kinematics motion spaces, specifically for RM joint angles $\theta_{3},\theta_{4},\theta_{5}$, and $\theta_{6}$ when translating linear motion from the A/DRC Pick Cylinder position to the RM home position.

### 3.2. Assembly Tasks Planning and STPN Model

#### 3.2.1. Planning Assembly Tasks

**Figure 8:**
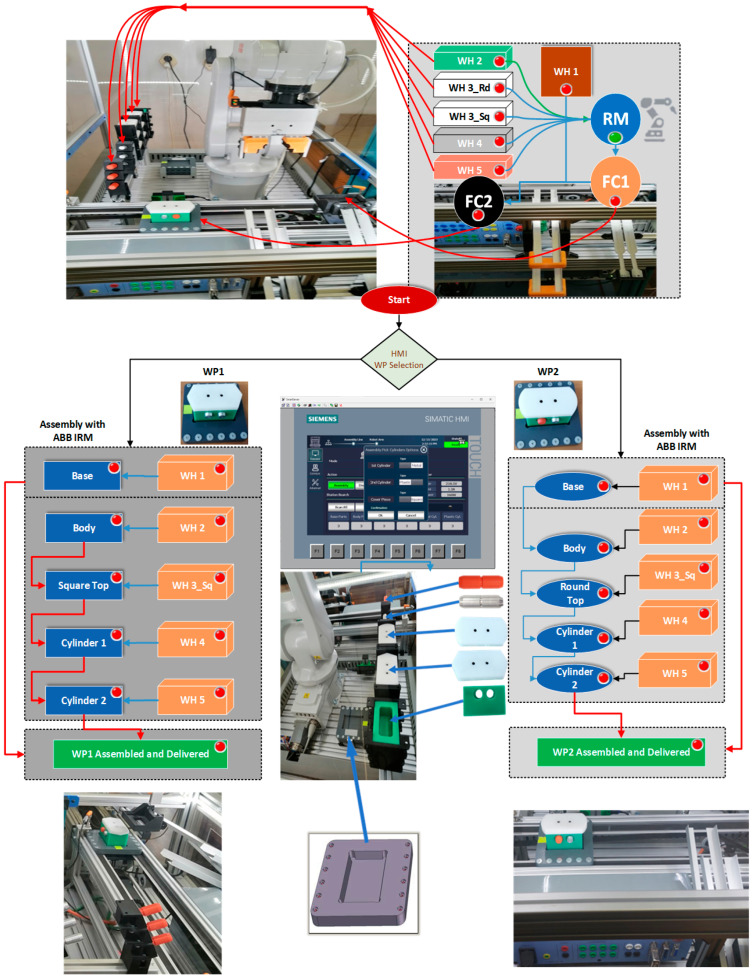
Node-RED task planning for the A/DRC assembly operation of WP1 or WP2.

A/DRC assisted by ABB 120 IRM performs assembly and disassembly operations of a predefined 5-component product for two workpieces called WP1 and WP2, [7,12,30]. The assembly flow process starts from storing and supplying subcomponents and progresses to the final delivery of the fully assembled, designated product type onto the cell conveyor unit, which functions as a transport line. The workpiece to be assembled consists of five components: a base pallet, a body, a top, and two inserted cylinders, which may be either plastic or metal. To introduce flexibility in manufacturing, two workpieces can be handled in the robotic cell depending on the operator’s selection. The top part is different, it can be either with square edges (WP1) or with a rounded shape (WP2). The assembly process involves RM performing repetitive tasks of picking, handling, and transporting the subcomponents to the specified assembly area within the cell, task planning for the assembly process of WP1 or WP2, is shown in Figure 8. First, the base pallet is placed on the conveyor FC2, then the product is partially assembled on a suspended bracket called Assembly Tray FC1. The body, top, and cylinders are fitted in this location, then the structure is placed on the base pallet. Once the assembly is complete, the assembled workpiece is transported via the conveyor FC2 to the right side of the cell, the delivery point for assembled products, serving as a starting point for another manufacturing process.

#### 3.2.2. STPN Model for Assembly

**Figure 9:**
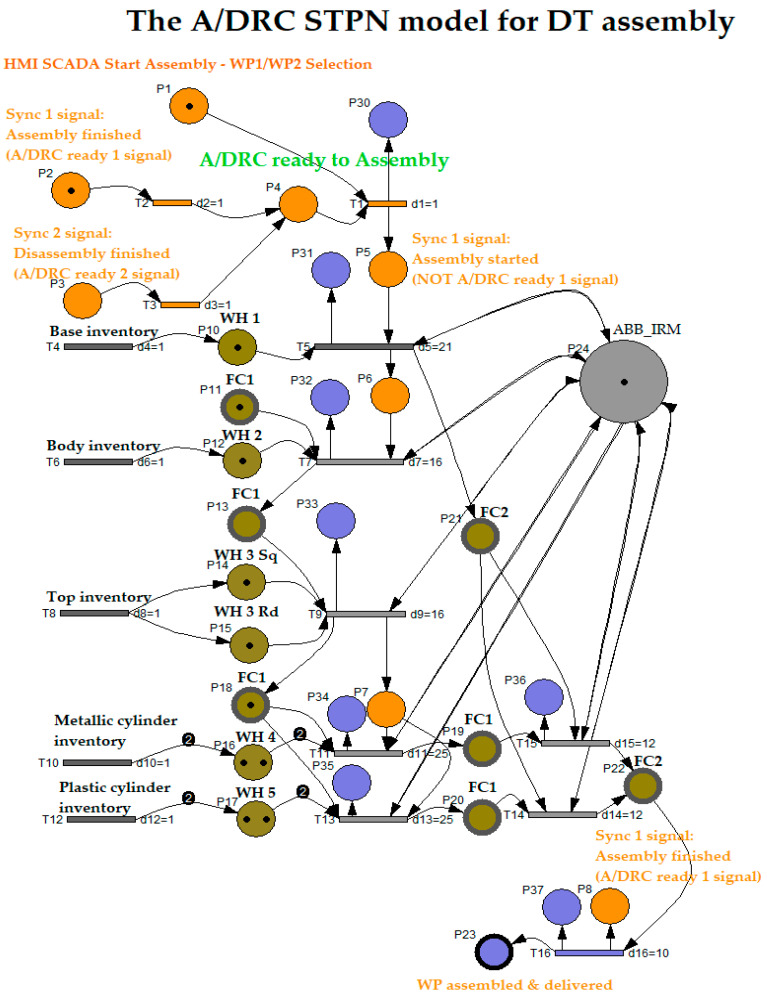
STPN model for the A/DRC assembly operation of WP1 or WP2.

STPN model for A/DRC assembly operation starts by selecting one of the workpieces WP1 or WP2 to be integrated per component by the manufacturing process into the final product. The assembly process involves RM picking the subcomponents from the storage units, handling and assembling them on the Assembly Tray FC1, and transporting the assembled workpiece on the conveyor FC2 to the right-side exit line of the cell, all this with the corresponding time durations. Synchronizing signals for assembly processes are required, for time-align and monitoring checks issues of the PLC program. Therefore, a Ready signal from previous operations like assembly or disassembly is a condition for starting the current cell task. The STPN model, simulated in Sirphyco [31] is shown in Figure 9.

The STPN is defined as follows:

(44) $$STPN_{A}=\left\{\begin{array}{ccc} TPN_{A}, & E, & Sync \end{array}\right\},\;$$

where $TPN_{A}$ is the Timed Petri Net model and corresponds to the assembly function.

(45) $$TPN_{A}=\left\{P_{A},\;T_{A},{Pre}_{A},Post_{A},m_{0_{A}},tempo_{A}\right\}\;$$

$P_{A}$ is the partitioned set of assembly $TPN_{A}$ model places:

(46) $$P_{A}=\left\{P_{control_{A}},P_{assembly_{A}},P_{supervision_{A}}\right\}$$

$P_{{\mathrm{control}}_{A}}$ (Orange color places) represent the states of the PLC program functions control tasks:

(47) $$P_{{\mathrm{control}}_{A}}={\left\{P_{i_{A}}\right\}}_{i_{A}=\bar{1,8}}$$

$P_{{\mathrm{assembly}}_{A}}$ (Olive color places) are the discrete states of the flexible assembly tasks:

(48) $$P_{{\mathrm{assembly}}_{A}}={\left\{P_{j_{A}}\right\}}_{j_{A}=\bar{10,23}}$$

$P_{supervision_{A}}$ (Blue color places) are the states generated for cell monitoring and supervision of the important tasks during the flexible assembly process:

(49) $$P_{supervision_{\;A}}={\left\{P_{j_{A}}\right\}}_{j_{A}=\bar{30,37}}\;$$

$T_{A}$ is the partitioned transition set of assembly for $TPN_{A}$:

(50) $$T_{A}=\left\{{T_{CondForAssembly_{A}},T}_{ReadyForAssembly_{A}},T_{assembly_{A}},T_{assembl{ed}_{A}}\right\}$$

- $T_{CondForAssembly_{A}}$ are the discrete transitions acknowledging that no other operation is running (assembly or disassembly);
- $T_{ReadyForAssembly_{A}},$ transition for assembly conditions: SCADA assembly command is ON, and cell is Ready (no other operation is running);
- $T_{assembly_{A}}$ are the discrete transitions of the flexible assembly tasks;
- $T_{assembled_{A}}$ marks the final transition when the assembled workpiece is conveyed by FC2 to the right-side exit line of the robotic cell.

(51) $$T_{CondForAssembly_{A}}=\left\{T_{2},\;T_{3}\right\}\;$$

(52) $$T_{ReadyForAssembly_{A}}=\left\{T_{1}\right\}\;$$

(53) $$T_{assembly_{A}}={\left\{T_{i_{A}}\right\}}_{j_{A}=\{\;\bar{4,16\;}\}}\;$$

(54) $$T_{assembled_{A}}=\left\{T_{16_{A}}\right\}$$

**Figure 10:**
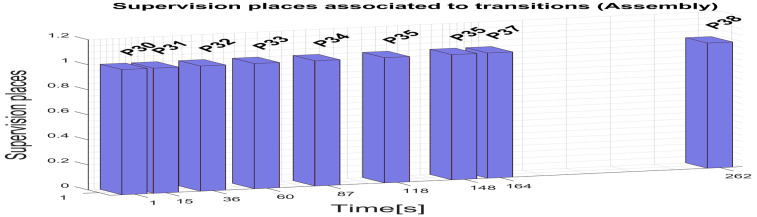
Sirphyco simulation of STPN model for the A/DRC assembly operation of WP1 or WP2.

For assembly on the robotic cell assisted by ABB 120 IRM, the supervision places (Figure 10) in the set (49) delegated to monitor the transitions set (50) are as follows:

- P30, T1 supervision transition is executed when the assembly task starts, and conditions are met, meaning the SCADA assembly command is ON, and A/DRC is Ready (no other operation is running);
- P31, transition T5 is executed when Base plate is placed on the conveyor FC2;
- P32, T7 supervision transition is performed when the Body part is placed on the Assembly Tray FC1;
- P33, T9 transition is carried out when assembly actions for choosing Top part with round or square edges corresponding to selected WP1 or WP2 to be manufactured;
- P34, transition T11 is executed when choosing the Metal cylinder for assembly;
- P35, transition T13 is executed when choosing the Plastic cylinder for assembly;
- P36, T15 transition is running when the assembled workpiece on the conveyor FC2 is transported to the exit line of the robotic cell;
- P37, T16 supervision transition is executed when the Assembly task is finalized in the cell and the Ready signal is set to ON.

$Pre_{A}$ is the input incidence function.

(55) $$Pre_{A}:P_{A}\times T_{A}\to Q_{{+}_{Apre}}\;$$

$Post_{A}$ is the output incidence function.

(56) $$Post_{A}:P_{A}\times T_{A}\to Q_{{+}_{Apost}}$$

$m_{0_{A}}$ is the initial marking set of tokens corresponding to Assembly places of the STPN modeled process.

$tempo_{A}$ is a function that defines the timings associated with the transitions.

(57) $$tempo_{A}:T_{A}\to Q_{{+}_{AT}}\cup \left\{0\right\}$$

E is the set of external events.

(58) $$E=\left\{Ev^{1},Ev^{2}\right\}\cup \left\{e\right\},$$

**Sync** application in definition (44) is a function from the set of discrete transitions corresponding to the assembly of A/DRC external events joined with the neutral element e,

(59) $$Sync:\left\{T_{2},\;T_{3}\right\}\to \left\{Ev^{1},\;Ev^{2}\right\}\cup \left\{e\right\}$$

The synchronization signal that generates the Ready signal when no Assembly task is running in the robotic cell:

(60) $$Sync\;1\text{\_}A:\;T_{2}\to \left\{Ev^{1}\right\}$$

(61) $$Ev^{1}=Sync\;1\text{\_}A\;\;signal$$

The synchronization signal that generates the Ready signal when no disassembly task is running in the robotic cell:

(62) $$Sync\;2\text{\_}A:T_{3}\to \left\{Ev^{2}\right\}$$

(63) $$Ev^{2}=Sync2\text{\_}A\;signal$$

### 3.3. Disassembly Tasks Planning and STPN Mode

#### 3.3.1. Planning Disassembly Tasks

**Figure 11:**
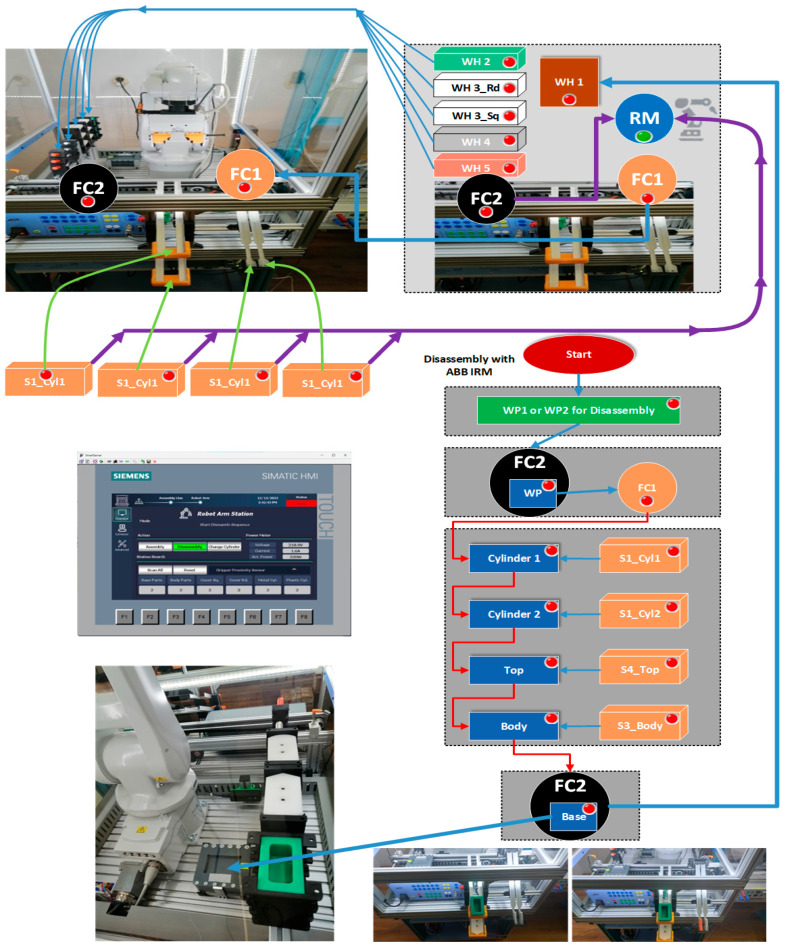
Node-RED task planning for the A/DRC disassembly operation of WP1 or WP2.

Workpieces ready for the disassembly process are delivered on conveyor FC2, on the right side of the robotic cell, serving as a “check-in” location point for products ready for disassembly. Further, the workpiece is transported by conveyor FC2 to the dismantling location where RM will do the rest of the disassembly process through repetitive tasks of picking and placing subcomponents in the reverse order as the assembly operation was performed. Cylinders, top, and body parts will be extracted and placed in the dedicated slide compartments on the cell, front bottom panel. The last task performed by RM is picking the base pallet from FC2 and placing it into the designated storage place W1, inside the cell. Task planning for the disassembly process is shown in Figure 11. The local control system-HMI is shown as well, for selection and starting the disassembly process.

#### 3.3.2. STPN Model for Disassembly

**Figure 12:**
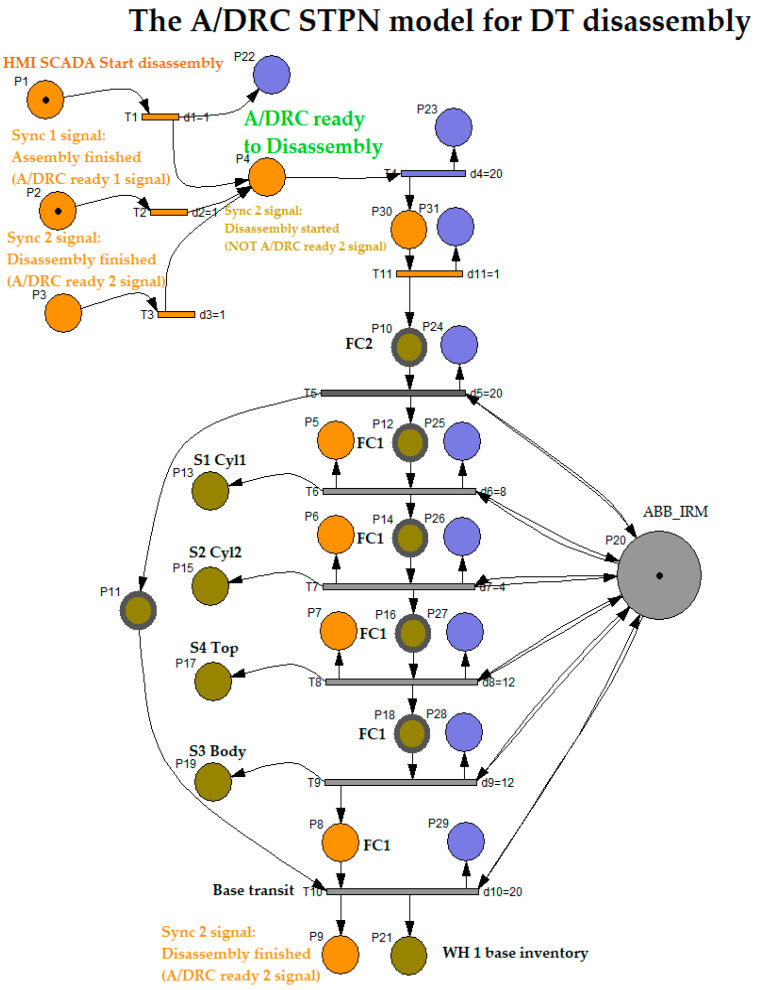
STPN model for the A/DRC disassembly operation of WP1 or WP2.

The disassembly process involves first that WP1 or WP2 workpieces will be delivered for disassembly process on the right side of the station and transported on the FC2 conveyor. RM picked and placed the structure in the Assembly Tray FC1 where all the subcomponents will be dismantled, then finally the last part is handled, and the base pallet will be stored on the dedicated storage unit. All these disassembly operations and transitions are executed with the corresponding time durations. The same two synchronizing signals, as for the assembly process are required, for time-align and monitoring checks issues of the PLC program. Therefore, Ready signals from previous operations like assembly or disassembly will be the starting condition for the disassembly task. The STPN model, simulated in Sirphyco [31], is drafted in Figure 12.

The STPN model for the disassembly process is defined by the following set:

(64) $$STPN_{D}=\left\{\begin{array}{ccc} TPN_{D}, & E, & Sync \end{array}\right\},$$

where TPN is the Timed Petri Net model, E is the external events array of transitions, and Sync is an application signals set corresponding to a set of transitions—conditions to be met, defined as external events involved in the disassembly process.

The TPN is a sextuplet:

(65) $$TPN_{D}=\left\{P_{D},\;T_{D},\;Pre_{D},\;Post_{D},\;m_{0_{D}},\;tempo_{D}\right\}$$

The elements of the Timed Petri Net model, $TPN_{D}$, corresponding to the disassembly function are as follows:

$P_{D}$ is the partitioned set of disassembly $TPN_{D}$, model places:

(66) $$P_{D}=\left\{P_{control_{D}},P_{disassembly_{D}},P_{supervision_{\;D}}\right\}$$

$P_{{\mathrm{control}}_{D}}$ (Orange color places) represents the states of the PLC program disassembly functions control tasks:

(67) $$P_{{\mathrm{control}}_{D}}={\left\{P_{i_{D}}\right\}}_{i_{D}=\bar{1,9}}$$

$P_{disassembly_{D}}$ (Olive color places) represents the discrete set of states, associated with the functions of the flexible disassembly tasks:

(68) $$P_{\mathrm{disassembl}y_{D}}={\left\{P_{j_{D}}\right\}}_{j=\bar{10,21}}$$

$P_{supervision_{D}}$ (Blue color places) are the states generated for robotic cell monitoring and supervision of the main tasks during flexible disassembly process for WP1 or WP2:

(69) $$P_{supervision_{\;D}}={\left\{P_{j_{D}}\right\}}_{j=\bar{22,29}}\;\;$$

$T_{D}$ is the partitioned transition set of assembly for $TPN_{D}$**:**

(70) $$T_{D}=\left\{{T_{CondForDisassembly_{D}},T}_{ReadyForDisassembly_{D}},T_{disassembly_{D}},T_{disassembl{ed}_{D}}\right\}$$

- $T_{CondForDisassembly_{D}}$ are the discrete transitions acknowledging that no other operation is running (Assembly or Disassembly).
- $T_{ReadyForDisassembly_{D}},$ is the transition for disassembly conditions: SCADA disassembly command is ON, and the cell is Ready (no other operation is running).
- $T_{disassembly_{D}}$ are the discrete transitions of flexible assembly tasks.
- $T_{disassembled_{D}}$ is the final transition when workpiece subcomponents are dismantled, transported, placed, and stored in their storage units.

(71) $$T_{CondForDisassembly_{D}}=\left\{T_{2},\;T_{3}\right\}\;$$

(72) $$T_{ReadyForDisassembly_{D}}=\left\{T_{4}\right\}\;$$

(73) $$T_{disassembly_{D}}={\left\{T_{j_{D}}\right\}}_{j_{D}=\{\;1;\bar{5,10\;}\}}\;$$

(74) $$T_{disassembled_{D}}=\left\{T_{10_{A}}\right\}$$

**Figure 13:**
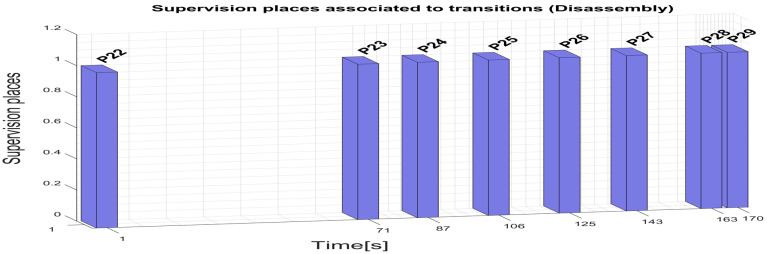
Sirphyco simulation of STPN model for the A/DRC disassembly operation.

For the robotic cell, WP1 or WP2 disassembly operations, assisted by ABB 120 IRM, the supervision places in the set (69) are delegated to monitor the transitions in the set (70). Supervision transitions modeled with STPN in Sirphyco, Figure 13, for the disassembly process of WP1 or WP2, are as follows:

- P22, T1 supervision transition is executed when the SCADA disassembly command is triggered;
- P23, T4 supervision transition is executed when the disassembly task starts and conditions are met, meaning the SCADA disassembly command is ON, and the cell is Ready (no other operation is running);
- P24, T5 supervision transition is executed when the workpiece to be dismantled is delivered on transport conveyor FC2, at a specific position to be handled by the ABB 120 IRM;
- P25, T6 supervision transition is executed when the upper part of the workpiece is placed on the Assembly Tray FC1, and RM is extracting the first cylinder;
- P26, T7 transition is carried out when RM is extracting the second cylinder;
- P27, T8 supervision transition is performed when the Top part is picked and placed by RM on the corresponding storage unit;
- P28, T9 supervision transition is executed when Body part is picked and placed by RM on the corresponding storage unit;
- P29, T10 supervision transition is executed when the Base part (Pallet) is transported by the conveyor FC2 to the right-side exit line of the cell, to be externally stored, the disassembly process is finalized, and the Ready signal is set to ON.

$Pre_{D}$ is the input incidence function.

(75) $$Pre_{D}:P_{D}\times T_{D}\to Q_{{+}_{Dpre}}$$

$Post_{D}$ is the output incidence function.

(76) $$Post_{D}:P_{D}\times T_{D}\to Q_{{+}_{Dpost}}$$

$m_{0_{D}}$ is the initial marking set of tokens corresponding to the Disassembly places of the STPN modeled process.

$tempo_{D}$ is a function that defines the timings associated with the transitions.

(77) $$tempo_{D}:T_{D}\to Q_{{+}_{DT}}\cup \left\{0\right\}$$

Same as in the Assembly process, the **Sync** application in definition (64) is a function from the set of discrete transitions corresponding to assembly and disassembly external events:

(78) $$Sync:\left\{T_{2},\;T_{3}\right\}\to \left\{Ev^{1},\;Ev^{2}\right\}\cup \left\{e\right\}$$

The synchronization signal that generates a Ready signal when no assembly task is running in the robotic cell:

(79) $$Sync\;1\text{\_}D:\;T_{2}\to \left\{Ev^{1}\right\}$$

(80) $$Ev^{1}=Sync\;1\text{\_}D\;\;signal$$

The synchronization signal that generates an A/DRC Ready signal when no disassembly task is running in the robotic cell:

(81) $$Sync\;2\text{\_}D:\;T_{3}\to \left\{Ev^{2}\right\}$$

(82) $$Ev^{2}=Sync2\text{\_}D\;\;signal$$

### 3.4. Three-Dimensional Virtual Model Implementation Using Siemens NX MCD

**Figure 14:**
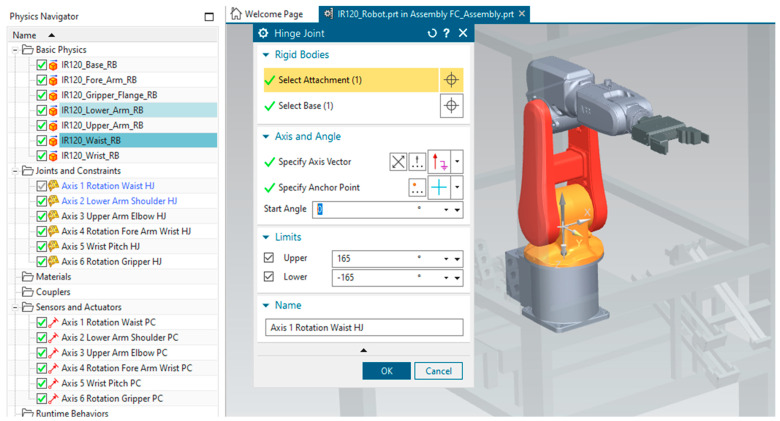
DT 3D virtual model design in NX Mechatronics Concept Designer. Mapping the properties and behavior of virtual components to external signals.

In this paper, 3D modeling and dynamics of the A/DRC including robot kinematic motion is developed in Siemens NX MCD [13], starting with the ABB 120 IRM model, developed in CATIA CAD Tool (Figure 14). The rest of the integrated mechatronic equipment is also digitally modeled like the conveyor belt, gripper, storage units for each of the final product components, and the housing of the complete setup. In this 3D model project, we use specifications and models from manufacturer catalogs to accurately replicate and mirror the physical layer. This approach ensures a consistent representation of the machines in the factory, aligning their dimensions, physical properties, and kinematic behavior with the actual mechatronics equipment [32,33]. Three-dimensional model representation, as an important part of the Digital Twin (DT) technology, must be converted to a proper simulation model that interacts with the twins, using specialized software tools, by integrating mechanical and software components into a unified simulation environment [1,2].

These include product lifecycle management (PLM) platforms, such as Siemens NX used in this research, which integrates Siemens Digital Industries Software products, or other 3D factory simulation software solutions for manufacturing and robot offline programming (OLP). MCD is an application inside Siemens NX that allows the simulation and design of kinematic models using external signals based on CAD files. The physics-based 3D model, utilizing Digital Twin (DT) technology, can be controlled and simulated in real-time with the actual PLC through Hardware-in-the-Loop (HIL). This approach enables early verification of the code and identification of potential issues for engineering process control program validation. As shown in Figure 14, the 3D model in NX MCD design starts with creating Solids and, subsequently, a Rigid Body feature is assigned to each part. For that, physical characteristics are assigned as well like weight, center of mass, and inertia, which influence the body dynamics in the simulation. Further Collision Body proprieties are assigned to motion graphical elements, to be able to have physical interaction with the other objects. Machine motions and dynamic interactions between components are applied for each of the 6 robot axes ${(\theta }_{1},\theta_{2},\theta_{3}{,\;\theta }_{4},\theta_{5},\theta_{6})\;$. Therefore, NX MCD joint connection implementation is performed, to enable virtual kinematic movements [32], according to linked signals interfaced and calculated by the virtual controller. Furthermore, controls are defined for the physics simulation of the entire machine in the digital space, providing an interface for simulating kinematic model behaviors in response to PLC inputs. NX MCD allows communication protocols with external programs; in this project, the standard OPC UA interface is used for broader connectivity. The 3D virtual interface, NX MCD establishes communication over OPC UA with PLC. A separate MCD signal should be created for each IO Field variable, which enables the mapping of sensor and actuator signals to virtual components for precise testing.

## 4. Digital Twin Technology

### 4.1. Multilevel Architecture

**Figure 15:**
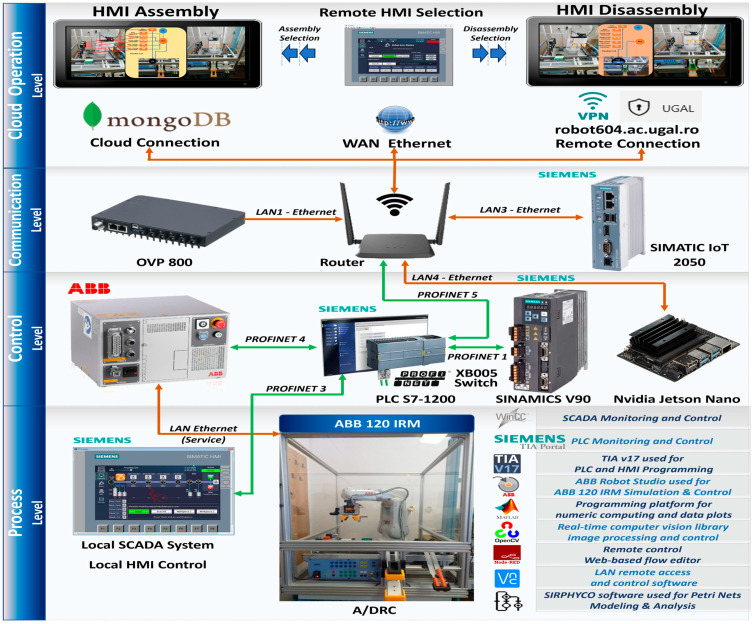
Cloud and VPN-based Control Multilevel Architecture enables hierarchical coordination of system components and organizing processes into multiple layers.

The multilevel control structure, features both distributed and centralized topology, all shown in Figure 15, consisting of the following equipment layers:

- Remote operation level, SCADA, and local HMI, for remote control of the A/D devices, for handling operations, and for real-time monitoring and data acquisition [14];
- Communication level handles data transfer and A/D tasks synchronization. SIMATIC IOT2050 has an important role in this hardware structure concept, as a bidirectional data transfer gateway for all edge devices with the Cloud platform [9];
- Control level with PLC S7-1200 which acts as a Central System that controls and synchronizes the overall A/D operations. Moreover, the PLC handles communication and program execution for all devices like ABB IRC5C IRB120 controller, with the Intelligent Siemens Servomotor Drives SINAMICS V90 and NVIDIA Jetson Nano—a small, powerful computer used for embedded applications, especially for Image-system processing solutions. The components detection task is performed by the OVP 800 video processing unit (VPU) with OpenCV-Python 4.4.0.44 open-source library [16];
- Process level, the robotic cell devices like: ABB 120 IRM with electric gripper, conveyor motors, storage, electrical and pneumatic actuators, all IO Field sensors, and HMI KTP 700 as A/DRC local visualization and control system.

### 4.2. PLC Software Design

**Figure 16:**
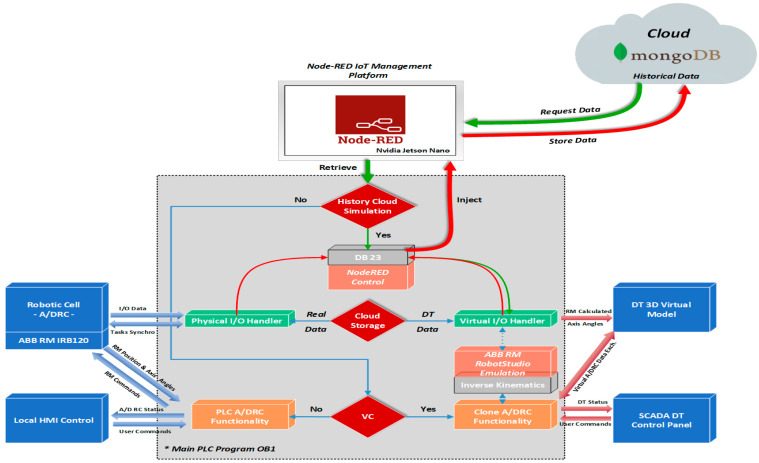
Siemens PLC Main program OB1 functionality chart and structure.

Starting from the main idea of building a virtual replica of the physical process and behavior, a PLC project is designed in the Siemens TIA Portal to simulate, monitor, and optimize its performance before software deployment and onsite commissioning [34]. Moreover, additional features can be added, like integration with IoT devices or Cloud Storage data and History Cloud DT playback for tracking and diagnosing potential events or problems that can arise during normal operation [9]. Therefore, for developing an accurate virtual model, PLC software is mirrored, keeping the same logic and behavior as the physical system, but programmed to communicate via OPC UA with the twins—NX MCD 3D virtual model [13]. OPC UA is one of the most straightforward and efficient protocols for enabling communication between multiple Industry 4.0/5.0 and IoT applications, minimizing the potential for communication issues, in this project, PLC acts as an OPC Server. Additional software tools are integrated for model validation, simulating various scenarios and control strategies and detecting potential issues or software bugs. The physical system I/O handler providing robotic cell status from sensors, actuators, and ABB 120 IRM positions and axis angles are replicated within a separate PLC function block—referred to as a Virtual I/O handler, by evaluating the same control logic and sensor behaviors. When DT is active, the Virtual I/O handler is the middleware for I/O data sent and received by the virtual model, as seen in Figure 16.

ABB RobotStudio functions [19], dealing with operations of A/DRC are also emulated in the main PLC, generating the designated motion angles and positions through inverse kinematics implementation and sent further to Virtual I/O Handler. The core function that implements the analytical method algorithm for computing the joint values ${(\theta }_{1},\theta_{2},\theta_{3}{,\;\theta }_{4},\theta_{5},\theta_{6})$ of the ABB 120 IRM is the inverse kinematics function block. Function blocks for inverse kinematics calculation, motion planning, and path generation, along with the software integration, communication with Virtual I/O Handler, and operation within the DT model, are developed in Siemens TIA Portal V17 [34]. The design aims to create a reusable framework applicable to other PLCs. While modern PLCs feature more efficient processors and expanded memory, implementing and designing a 6-DOF robot inverse kinematics calculation remains an uncommon task in PLC programming and engineering. This is because analytical methods for determining joint solutions based on the robot end effector’s position require intensive mathematical computations, particularly higher-order matrix operations.

**Figure 17:**
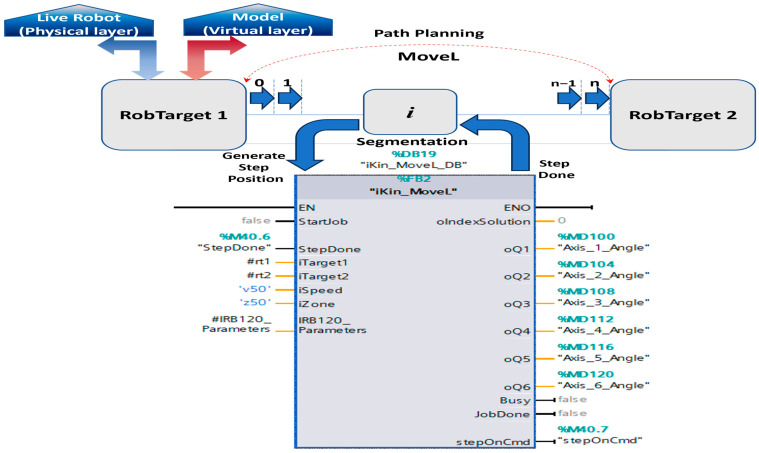
PLC function block design algorithm for MoveL linear motion solution through path segmentation.

These demands challenges related to PLC-specific issues, such as execution cycle time limitations and expertise level for code optimization. The designed program calculates and returns a single joint angle solution based on the robot arm’s position, provided as input from the ABB RobotStudio emulation software module, keeping the standard ABB *robtarget* data type format for the position. Based on the SCADA user input selection, whether it involves visualizing the robot’s live parameters or real-time process data, opting to model and simulate various A/D operations with different cycle times, analyzing and comparing performance, or identifying potential issues, the PLC consistently receives the end effector’s position as an input. Based on the provided linear speed parameters, a separate function block handles path planning and motion control by segmenting the path between robot targets and interpolating the positions. The function block responsible for path planning and controlling linear motion (using the MoveL command) from the initial robot pose to the final goal position and orientation is shown in Figure 17. It employs a smooth function, known as Cubic Polynomials, when the desired velocities at intermediate points are non-zero and is an efficient way to interpolate between two points in space, ensuring that both position and velocity constraints are met.

**Figure 18:**
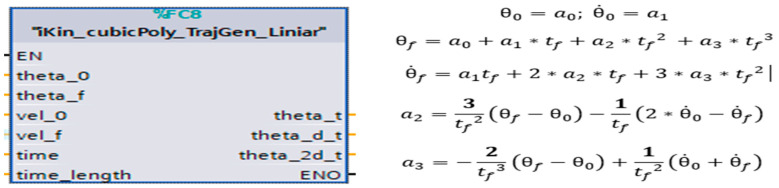
Siemens PLC function block for Trajectory path generation implementing Cubic Polynomials smooth function.

Cubic polynomials is a commonly used method in robotics for smooth path planning, particularly in applications requiring continuous motion and velocity profiles (Figure 18). This method ensures efficient and precise control of robotic systems, balancing simplicity with effective performance in many practical scenarios. A separate robot position is generated for each timestamp. i out of n time segments, which are then used as input for each calculation step of the robot joints through the inverse kinematics algorithm. A watchdog was implemented to ensure smooth graphical movement at each timestamp during the robot’s motion between two targets.

This guarantees stable, real-time communication, and rapid data reception, preventing error handling and unintended manipulator movement [6,29]. Out of the eight generated solutions for the inverse kinematics, a single solution is selected by computing the best fit that complies with the mechanical limitations and physical characteristics of the ABB 120 IRM. This solution is chosen based on the target configuration quadrant for axes 1, 4, and 6, along with the ABB *robtarget* data type.

Three conditional execution controls are implemented in the main PLC program OB1, as an option that determines if the specific instructions or module will be executed, according to operator choice, as seen in Figure 16: History Cloud Simulation, Cloud Storage Source, and Virtual Commissioning. In normal situations, with no user intervention in the cell functionality, the A/D operations are carried out/executed on the physical system. The DT is turned off, and essential robotic cell structured data, such as axis angles, positions, sensor statuses, actuator states, and various process stages, are captured and stored in a JSON-like format in the Cloud. Cloud is used to collect, process, and store data for real-time analysis and long-term storage [9].

In this project setup, the MongoDB Cloud platform is selected for its flexibility, scalability, and ease of use, making it an ideal solution for virtually mapping the entire physical process. The calculated virtual data can be stored in the Cloud, instead of real process data, when the DT system is running, this option is selected from the SCADA DT Panel. By reading the Siemens PLC DB23 process live data, Node-RED is used to store data in MongoDB offering a powerful, flexible, and scalable solution for handling data from both sources [15].

Virtual Commissioning option is also integrated into the system by testing Clone PLC functionality against the real function block programs. Sensors from the physical system are integrated into the digital model allowing real-time data to be used for the simulation. The real physical I/O handler sensor data can be fed into the virtual system, enabling the simulation to reflect real-world sensor behavior. Therefore, the readings from the conveyor and ABB 120 IRM can be compared to the calculated values data in the DT to ensure consistency and accuracy. History Cloud Simulation is used to replay and simulate past scenarios and robotic cell system operations based on real-world data collected over time, often using data stored in the Cloud. The process data, along with the robot’s state, axis angles, and positions are logged and can be monitored at each moment during the motion path segmentation. This simulation can be used for various purposes, such as performance analysis, predictive modeling, system optimization, and event failures to better understand process behavior and make more informed decisions for the future.

### 4.3. DT Communication, Synchronization and Control

**Figure 19:**
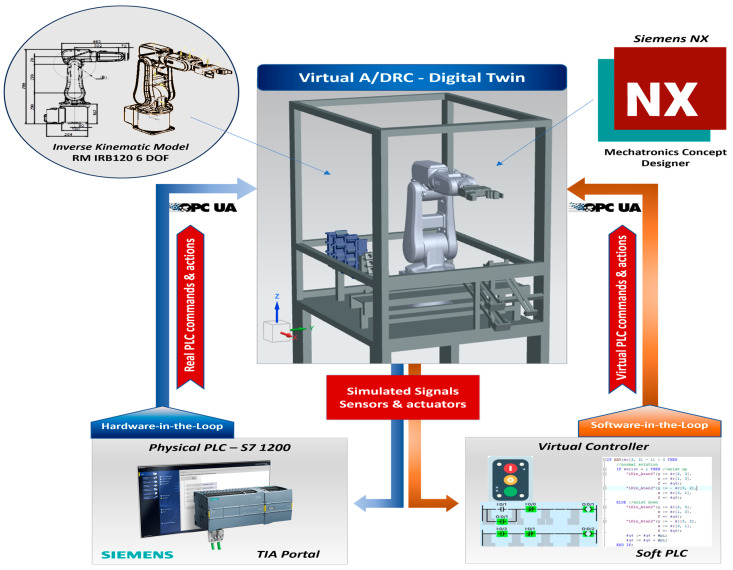
Hardware-in-the-Loop (HIL) Virtual Commissioning Concept.

For building the 3D virtual interface, to be able to connect the PLC in an HIL configuration, NX MCD establishes communication over OPC UA [13], therefore a Signal Mapping table is required for cross-reference connection with external signals, configured in the OPC UA Server from PLC. The OPC interface supports mapping with the IEC61131-3 language-PLC-specific protocol and offers a wide range of use and connectivity. Moreover, OPC UA is more and more preferred as a standardized protocol to transfer data between different levels of automation. Communication is performed in real-time, and information can be transferred between client and server and from server to server, the minimum update time for OPC is approx. 100 ms. When using a virtual controller in a Hardware-in-the-Loop configuration seen in Figure 19, a physical PLC is needed to synchronize and run the simulation with the same internal clock speed as the rest of the system, avoiding communications latency between the PLC and emulation software [35]. The primary objective of this study is to develop a Digital Twin application by implementing a simulated inverse kinematics model using MATLAB/Simulink. Subsequently, the data will be manually transferred from the MATLAB workspace to the A/DRC controller (PLC) for further processing and back from the virtual model to MATLAB. For future work, we plan to establish a communication link to adjust model parameters in real-time, transmit various A/DRC state information for processing, and read the updated information back into the system to enhance the simulation’s accuracy and adaptability.

Further, PLC software design also provides a new approach, besides the logic for the robotic cell and RM automation control—known as the physical layer, it provides communication interface links through PROFINET IO to SCADA and HMI [14]. For linking PLC with the DT, an extra gateway is used, SIMATIC IOT2050, providing IoT support for data to be transmitted to the Cloud and can exchange data between physical and virtual models, providing data transmission and data aggregation, in case is needed. It is well used for converting a range of communication protocols, due to specific features like its capability of Remote accessing I/O’s (Digital, Analog) and expanding with different types of smart sensors or Edge Devices [9,36].

### 4.4. Virtual Commissioning Concept; Hardware-in-the-Loop (HIL) Simulation

Aligning with the current trends in manufacturing, the integration of twins in the engineering process is accelerating mainly due to Industry 4.0/5.0, bringing these “smart” manufacturing systems capabilities into the digital world. As part of DT technology, the VC tool concept and technique is used, offering the potential to minimize the considerable delays and expenses typically involved in the challenging process of classical commissioning, system integration, and operation with (PLC).

The presented manufacturing technology allows the integration of the VC concept by taking advantage of the integrated bi-directional data exchange flow between the dynamic digital representation and the physical system. To connect the A/DRC hardware controllers with the NX MCD 3D model virtual plant, mainly the HIL simulation strategy is adopted, implemented, and performed as a Soft Commissioning approach, by testing the designed PLC programs against the physical PLC (Figure 19). By using the real physical controller, an HIL configuration offers the advantage of delivering the same performance as it would run in actual production by testing the hardware in all situations to ensure it can succeed in all scenarios as programmed. The same happens when there is a big software or hardware change; it ensures that everything still works the way it is intended. As mentioned, a key requirement for testing VC strategy setup is that the virtual factory model must behave exactly as the real model.

In what concern is HIL implementation for virtual plant models to work in tandem with PLC, since simulation has a specific, separate internal clock, this must be synchronized with the real, onsite PLC, for tasks to operate and for time-dependent functions to remain valid. For the implemented DT, specific functions are designed for PLC to be manipulated, synchronized, and run at the same speed as the rest of the system without affecting the test performance. HIL focuses on the PLC software that operates these systems.

Its primary benefit is the ability to identify errors at the initial stages of the factory implementation process, thereby reducing the need for extensive debugging during the physical commissioning phase. A set of PLC function blocks are implemented for the kinematic computation, from MATLAB simulation to PLC code, for digital representation of the complete engineering process.

## 5. Remote Monitoring and Control

### 5.1. Digital Twin Control Panel—SCADA

**Figure 20:**
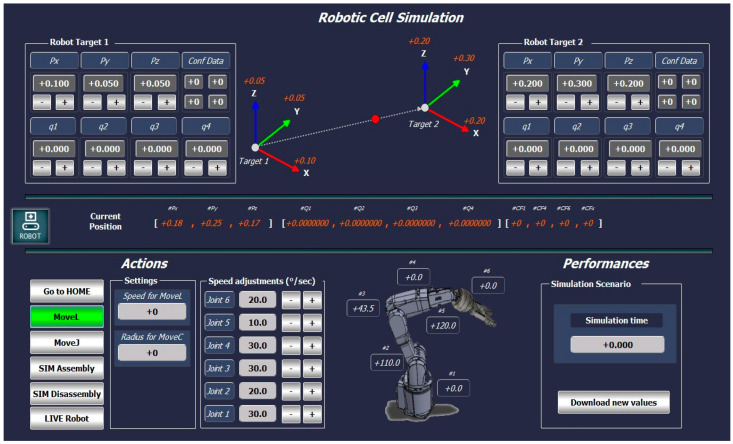
SCADA DT control panel of the A/DRC operations and RM motion control.

In this research, for VC implementation of the A/DRC physical process, beyond the 3D digital model representation, there is a need for a graphical user interface (GUI) tool. This visualization tool, serving as a DT operator control panel, is essential for interacting with the DT, it refers to a seamless two-way, bidirectional automated data exchange interface between physical and digital objects. It facilitates data monitoring, analysis, and comparison, as well as signaling and processing various events. Additionally, it supports simulation time reporting, user interaction, and setup control. SCADA also allows the users to manage data and control different scenarios or operations within the DT platform, including custom mimics—interactive graphical displays, specially designed for testing the calculation of the inverse kinematics, DT interaction, and process representation based on live and history Cloud data. These mimics enable the user to execute and test various functions in the DT virtual model for centralized control and management of different hypotheses and scenarios. This enables testing and diagnosing potential operational lags or calculation errors and identifying performance issues or slowdowns in the engineering process. As seen in Figure 16, by using the computation resources for PLC code execution (as described in Section 4.2) users can take advantage of the visualized interactive data regarding the inverse kinematics calculation by entering the end effector robot position given as an input, all the eight possible solutions are displayed on the screen. The rotation matrix is visualized in SCADA. Based on the operator’s selection, clicking the Simulate button allows the robot’s movement calculated set of angles to be visualized on the 3D model within NX MCD. The user can monitor and analyze real-time DT readings, at each step of the robot’s movement, starting from the given position to the final target, including linear translation path segmentation and inverse kinematics calculations (Figure 20). Robotic cell performance parameters can be monitored on this mimic, by setting different linear motion velocities or robot axis speeds, allowing various operations to be tested, including using Live Robot data as part of the Digital Shadow concept within DT technology. If a test scenario meets the required performance criteria, the validated speed parameters can be downloaded by the operator into the PLC. Historical Cloud Data are also presented, and upon request a target event is made available and is entered and sent further for processing in Node-RED for building the necessary Cloud interrogation queries [37,38]. Furthermore, the retrieved data are plotted on the screen. The major timeline spikes are visualized from the 180 seconds History interrogation query time and can be sent for playback in the DT 3D Model representation in NX MCD visualization.

### 5.2. IoT-Cloud-Based Control and Data Acquisition

The purpose of the presented architecture features the IoT Cloud connection for collecting all IO Field sensor data and enabling database storage and analytics for integrating the processed data into the DT application, for virtual historical and real-time process presentation [38]. Using an IoT-Cloud-based DT online monitoring system brings important features for the end user, regarding process information and online remote control of the A/DRC tasks using cutting-edge technology to monitor and detect abnormal behavior and equipment failure using model-driven HIL simulations. Moreover, Cloud storage providers offer robust security features, including encryption, access controls, and compliance with industry standards, especially ISO 27001 [39], regarding information security management systems [1,9,36].

**Figure 21:**
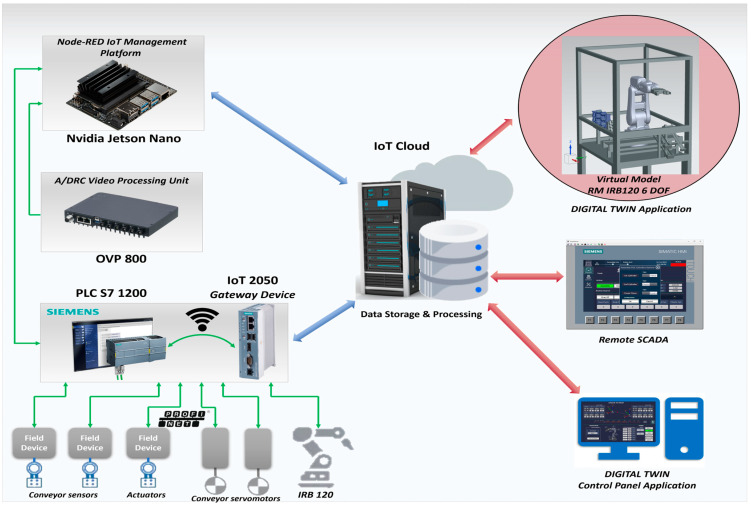
Overview of the general system setup for A/DRC model-driven digital twin (DT) framework using IoT Cloud-based Control and Data acquisition.

The proposed framework for remote monitoring and control of the A/DRC via the model-driven DT using IoT consists of the following components (Figure 21):

- IoT sensing device;
- Embedded computer NVIDIA Jetson Nano. Acts as a Node-RED IoT interface and Docker Engine client-server application. Remote connection is also available through dedicated VNC-Compatible Free Remote Desktop Software [37];
- ABB IRC5C controller. Executes the user-configured program for the industrial robotic manipulator of the robotic cell and communication to PLC;
- OVP 800 Video Processing Unit. For handling the station HD Cameras, through which the scanning process is performed and the configuration of the workpieces to be assembled is identified [16,17];
- Siemens IOT2050. An IoT gateway device, which enables the bidirectional communication of the PLC with the IoT Cloud platform over a wireless connection;
- Siemens PLC 1214C type. Acts as the main control unit, sends and receives data from all the robotic cell devices including LAN-connected servo drives, and process sensor data, and is responsible for synchronization of the A/DRC tasks. Data are further available for SCADA systems, Cloud storage (MongoDB), and DT applications.

Locally, at the operation level, by using the station HMI or on the Remote SCADA application, the user can control and simulate the process A/D operations, as well as monitor all the signals, sensor values, calculated data, and RM motion positions and angles transmitted by PLC on the screens [14]. Additionally, by using the onsite ABB touch pendant, the operator can execute the actions configured by users in the ABB IRC5C controller and program in RobotStudio [19].

**Figure 22:**
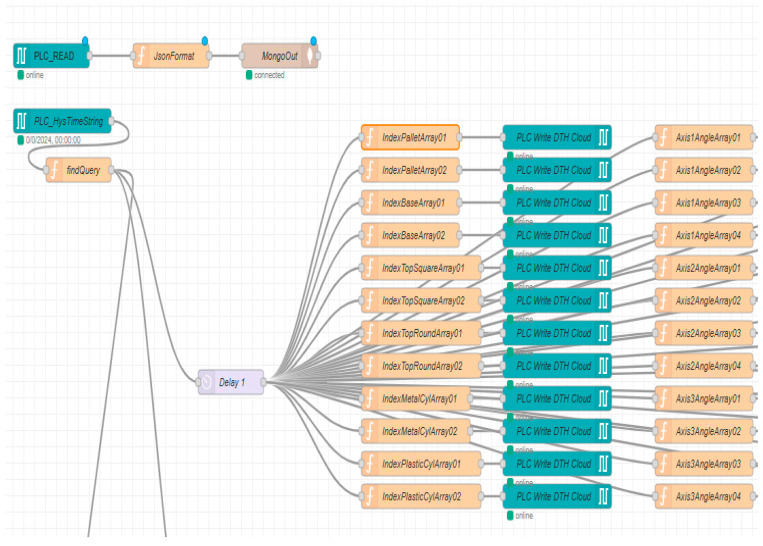
Diagram of the Node-RED and Cloud-PLC flow interaction.

The video processing unit, OVP 800, by running ROS2 Humbles camera management, controls the three IFM Cameras video streams through the IFM3D_ROS2 library. It facilitates data exchange with the PLC and integration with Node-RED hosted on Embedded computer NVIDIA Jetson Nano which acts as an IoT gateway and Cloud middleware device [15,37] (Figure 22). By integrating the video processing unit connection with the Jetson Nano, OVP 800 acts as an IoT sensing device. The Jetson Nano-embedded computer can send all the image processing data, robotic cell events, and sensor data to the IoT Cloud platform over a wireless Internet network connection for storage and for further vision data processing [16,17]. Siemens IOT2050 is a cutting-edge platform ideal for developing IoT smart applications and automating various industrial processes, designed for efficient IoT data management and communication [36]. In this paper, DT allows the integration with cell devices and sensors, sending/receiving data to/from PLC, enabling real-time data collection, processing, and control via the Cloud platform with enhanced connectivity features (Figure 20).

Data collected on the Cloud platform (MongoDB) can be further manipulated for advanced processing, prepared for analysis and presentation, for building different IoT Cloud applications such as remote SCADA for monitoring and Data analysis Server and, moreover, for receiving an earlier robotic cell timeframe data set for playback in DT application [9]. Cloud storage (MongoDB) receives real-time data from A/DRC via Node-RED, capturing cell status parameters essential for retrieving historical event data upon request from the DT. The following live A/DRC parameters are continuously stored: RM joint axis angles, RM current position, storage status (Pallet, Base, Top, Cylinders), RM status, conveyor status, A/DRC status, and emergency stops.

Network connection reliability for Wi-Fi and the Internet has a critical role; therefore, in the event of a Wi-Fi connection failure, IoT capability and functionality for Cloud-based Control and Data acquisition is affected, no data transmission is accomplished, and only DT warning messages and Cloud reporting services will be pushed in the application message viewer.

### 5.3. Remote HMI VPN Control

The proposed system uses two SCADA applications, one locally and one remote for monitoring and control of the A/D operations on a robotic cell process, equipped with an ABB 120 IRM. The structure and functionality of the entire system are based on a multilevel, decentralized architecture, integrating IoT, smart manufacturing, and intelligent digital technologies, like the VC technique in the context of Industry 4.0/5.0. Therefore, authorized users can safely monitor and control the cell process from anywhere, providing context that will help operators comprehend and predict potential unwanted events by delivering effective situational awareness. Moreover, by implementing and programming the robotic cell 3D virtual model A/D operations and task planning, including the ABB 120 IRM motions, remote SCADA acts as a DT operator panel for process simulation, data collection presentations, and analysis, with remote operation capabilities via Cloud or VPN [9,35,40,41]. The DT platform allows the complete management and control of the A/DRC processes and devices, with the possibility of customizing the simulations according to specific needs. In this paper, an IoT Cloud-based control and remote access application is implemented. Cloud Remote access allows operators to intervene on the robotic cell in a fast and secure way, reducing onsite interventions, and saving time and resources. A third-party Cloud software platform is required, Mongo DB, to interconnect IoT devices, Cloud computing, and storage to dynamically access remotely, for visualization and monitoring in SCADA, for analysis or prediction. MongoDB also provides additional features, like integrating with other Cloud applications or Reporting Services for data visualization [38]. Node-RED is a programming tool for dealing with IoT devices typically used for connecting different nodes, APIs, and online services to build a powerful smart IoT integrated automation system. The Node-RED server running locally on the Jetson Nano IoT device provides Secure Remote access and visualizes the robotic cell stored data on the Cloud. A simple and intuitive operational interface is developed for remote monitoring of A/D operations and controlling all IoT devices, for security configuration, reports, trends, and alarm acknowledgment. Accessing the Node-RED dashboard over the Internet through VPN connections provides an enhanced security environment by using Industry-Standard Encryption like Secure Socket Layer (SSL) and Transport Layer Security (TLS) [36]

## 6. Robotic Cell Real-Time Results and DT Virtual Model Validation

This research presents the design of an assembly/disassembly robotic cell application integrated with a Digital Twin for remote monitoring and control. It operates within an HIL simulator system, enabling testing, real-time process mirroring and monitoring, predictive maintenance, and remote operation. The system supports various tasks requiring the integration of intelligent digital technologies and Industry 4.0/5.0 concepts, such as Virtual Commissioning (VC), the Internet of Things (IoT), and Cloud computing.

Furthermore, the proposed Digital Twin (DT) serves as a crucial link, a bridge between the real and virtual worlds, enabling seamless interaction and data exchange, allowing monitoring, controlling, and optimizing of the physical processes in real time.

The control structure in the real world, serving as the counterpart to the Digital Twin, features a hierarchical yet multilevel and decentralized architecture. It is designed for supervising, monitoring, and controlling processes, executing operations, and planning assembly/disassembly (A/D) tasks. By continuously synchronizing with its physical counterpart through sensors and data analytics, allows the optimization and flexibility of A/D operations, predictability, and process efficiency.

In this paperwork, STPN modeling and simulation of A/D operations are integrated into a real-time application through SCADA and DT HIL platform. This is achieved by interfacing them with synchronized signals collected from the real process through PLCs and sensors. To evaluate and validate the DT model environment against the A/DRC real mechatronic process assisted by ABB 120 IRM, several tests and analyses were performed.

**Figure 23:**
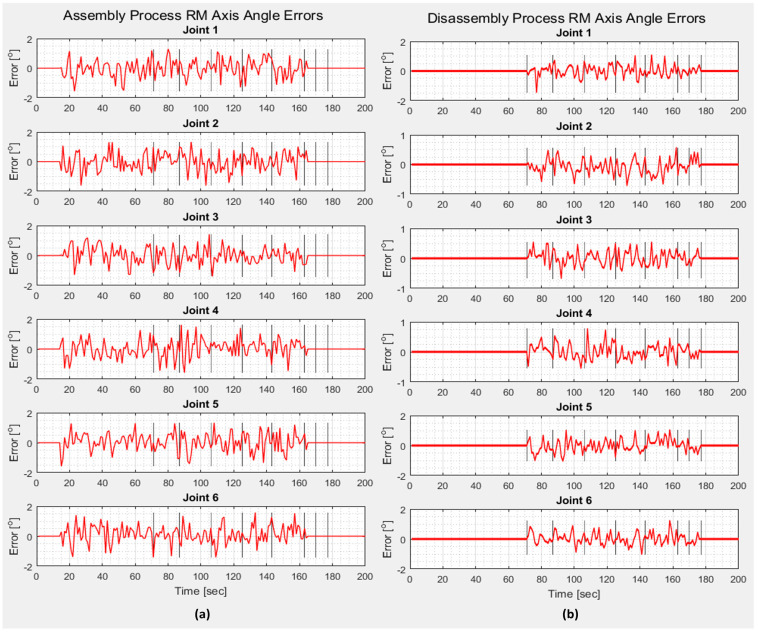
RM axis angles errors comparison for A/DRC process tasks according to STPN model transitions for physical system and DT virtual model: (**a**) A/DRC assembly operation; (**b**) A/DRC disassembly operation.

After implementation, the results of real-time control are presented and compared with the monitoring signals obtained from the DT IRM, which emulates movements specific to assembly and disassembly operations (Figure 23).

A/DRC assembly/disassembly operations involve repetitive tasks of IRM picking and placing subcomponents from the storage units, for handling and further transporting the workpiece on the A/DRC conveyor, all this with the corresponding time durations. For each IRM task, a MoveL motion command is performed, a linear movement from an initial position to a desired final position, a command specific to the ABB RobotStudio hardware controller. A function that emulates linear movements is also designed, implemented, and tested for DT IRM. By comparing the results for a single MoveL motion command from home to a desired position, the proposed virtual model emulated motion demonstrates high accuracy in path following, closely matching the real world. It also provides a reliable approximation for robot tracking, positioning, and joint axis angles.

These movements are simulated as well in Sirphyco, reflecting the discrete states of the corresponding STPN models. Comparing the results from the Sirphyco simulation of STPN models, slight differences were identified between the simulated data and the monitoring signals provided by PLC, which correspond to the real-time execution of control programs. Thus, the virtual digital component of the robotic cell manufacturing technology is validated in real time.

Following digital model development and running simulations, real-time implementations are integrated within the digital environment for validation and testing, where the results from the physical system are cross-referenced with simulation outputs (Figure 23). DT model slight differences were found, originating from PLC inverse kinematics algorithm calculations, especially from rounding the huge expressions of the rotation matrices and conversions, as well as some communication delays generated from data communication links, but these errors tend slowly to zero and do not affect the overall setup performance or bring behavior difference (Figure 23).

The robot’s virtual model and behavior were able to accurately replicate and follow with a high degree of reliability the A/DRC real mechatronic process, assisted by the ABB 120 IRM, to be as close as possible to the real industrial world.

## 7. Discussion

This research paper was undertaken to build an HIL DT framework with Cloud solutions and Remote VPN capabilities for a robotic cell assisted by ABB 120 IRM, based on a PLC implementation for an RM inverse kinematic virtual model.

The Digital Twin model is used to virtually replicate the physical robotic cell structure and represents an approximation of the real behavior of the A/DRC manufacturing process, enabling real-time monitoring, simulation, and optimization of processes for improved efficiency and performance.

Although the A/DRC functionality is designed for a specific manufacturing process, handling multiple assembly and disassembly operations for a predefined five-component workpiece, the PLC-based hardware and software DT design architecture is flexible and can be easily adapted to more complex manufacturing processes. As previously mentioned, the fundamental design concept of the hardware and software HIL DT design architecture integrates three major subsystems, which can operate, communicate, and synchronize with each other: PLC, SCADA, and DT 3D virtualization of the process using Siemens NX MCD.

The S7-1200 PLC is programmed using the Siemens TIA Portal to control both the physical and virtual A/DRC models within an HIL structure. If, at the real-time operation level, the automation control of the A/DRC manufacturing process is designed for a specific set of tasks—modeled and simulated using STPN models—the NX MCD virtual plant model implementation remains easily adaptable to a different robot manipulator or even an entirely different assembly/disassembly robotic cell process. The PLC program is designed for the kinematics of a six-axis industrial robot manipulator, with analytical methods for inverse kinematics calculation and implementation structured as modular PLC functions. These functions are generally applicable to any six-DOF robot manipulator by specifying its product details and characteristics. In this research, within the mechatronic laboratory robotic cell, the A/D tasks are executed by the ABB 120 IRM, with its specifications assigned as internal PLC variables.

Remote and local SCADA systems serve as the operator control panel for interacting with the Twins, integrating key functions such as real-time monitoring, control, and visualization of A/D tasks, as well as managing data and controlling different scenarios or operations for the DT with the HIL platform. An intuitive and interactive operational interface is developed in SCADA, primarily used for testing the calculation of the six-DOF robot manipulator’s inverse kinematics. The remaining graphical displays are dedicated to DT interaction, providing a live representation of A/DRC runtime data. These displays provide also support for Cloud data collection, presentation, and analysis through Remote VPN, integrating IoT for dynamic remote access, analysis, or prediction. The SCADA environment used in this project as the DT Operator Panel is highly flexible and can be configured to suit a variety of industrial applications and manufacturing processes. Additionally, it supports a wide range of communication protocols such as PROFINET IO, OPC UA, and MQTT, enabling connections with various third-party hardware and software. While it is currently implemented for a single robotic cell with a six-DOF RM, it can be easily deployed, modified, and adapted for large-scale operations involving multiple robotic cells in a variety of industrial applications.

The DT environment uses Node-RED, a powerful and highly adaptable open-source, flow-based development tool for smart IoT-integrated automation systems. It runs on the Jetson Nano IoT device, enabling secure remote access and visualization of the robotic cell’s stored data in the Cloud. Moreover, Cloud provides global scalability, allowing access to data from any location worldwide.

It is important to underline that developing and employing a DT application brings also limitations to enhancing operational efficiency. We will generally address the DT limitations, but we will primarily focus on the potential limitations of the proposed approach:

- **DT virtual model accuracy and fidelity.** A high level of accuracy in the DT model is crucial for organizations aiming to optimize productivity and operational performance. Virtual models need some more extensively validated dynamics and response validation and task planning algorithms to accurately represent the physical counterpart. The proposed A/DRC DT virtual model implementation is based on the RM inverse kinematics model and not on the internal robot’s dynamics, which adds a limitation to the level of RM model accuracy;
- **HIL DT extensive development time.** It is important to underline the fact that DT implementation, interaction, and 3D virtual model design during product development require significant time, resulting in longer development timelines. Therefore, DT system implementation can have an expensive initial cost in designing and deploying into the real engineering process;
- **RM inverse kinematics analytical solution is limited to simple robotic cell designs.** This paper proposes an analytical solution to the inverse kinematics problem of a 6-DOF RM using a geometric approach that utilizes trigonometric relationships and vector algebra to derive joint angles. This method is highly efficient and accurate, providing precise joint configurations without approximation, making it suitable for real-time applications where calculation speed is crucial. However, it has some limitations, as it does not apply to more complex structures and configurations and can be sensitive to singularities;
- **Reliability and data quality.** Integrating the IoT Cloud-based data for DT technology in a laboratory system faces challenges and concerns about the quality of the data. Robotic cells generate vast amounts of real-time data; therefore, streams from IoT devices require advanced storage and processing capabilities. Reliability and data quality for faster automation processes and DT communication interfaces are vital, which restricts their performance and prediction accuracy in real-world applications. Low-latency communication between the physical robotic cell and its twin is crucial for real-time applications, and Cloud connection speed rate and interruptions can lead to inconsistent data, especially when using IoT and History Cloud. Data security policies, data verification, checksums, and encryption techniques are not a concern in the A/DRC laboratory setup. However, these procedures should be implemented in real-world manufacturing processes, where data collection and transmission depend on robust IoT ecosystems and face real challenges related to data complexity and cybersecurity threats.

New directions for future research and development have arisen, to overcome the above-mentioned limitations. Several key areas require further development in future research:

- **Integrating Intelligent Digital Technologies**, such as Artificial Intelligence and Machine Learning, along with enhanced integration with IoT ecosystems;
- **Advancements in system optimization and digital twin efficiency**. The development of more efficient Digital Twin implementations and algorithms for virtual model dynamics and response validation enhances accuracy, real-time performance, and system reliability in industrial applications;
- **Addressing the challenges in Industry 4.0/5.0**, such as the integration of collaborative robots into manufacturing systems, particularly within robotic cells, and their connection with DT technology. This is an area that requires further attention, designing robotic cells to ensure safe collaboration with human workers requires additional safety protocols, sensors, and programming;
- **Enhancing/Increasing complexity in robotic cell Integration with Digital Twins**. Adding layers of complexity in terms of data collection, processing, and response of the robotic cell requires advanced design, careful integration, and more sophisticated control systems to ensure the accuracy of DT models, efficiency, and reliability. Moreover, the complexity of a robotic cell in its design, operation, and integration with DT should be addressed, starting from adding multiple robots or adding extra tasks into a single robotic cell integrating more sophisticated control algorithms, and machine learning approaches, and combining with the implementation of VC techniques in this setup.

## 8. Conclusions

In this paper, we propose an HIL DT application of A/DRC, assisted by the ABB 120 IRM, with the following key contributions:

- **Multilevel architecture, hardware, and software setup;**
- **Addressing potential limitations of the HIL DT laboratory setup.** These limitations are related to the accuracy and fidelity of the virtual model, which impacts DT efficiency and production performance but can be improved through process optimization and predictive maintenance;
- **A/DRC STPN simulation for validation of model and control strategy based on task planning integration and synchronization**. This hybrid modeling approach ensures real-time adaptability and flexibility in production workflows, facilitating task synchronization and enhancing the system’s responsiveness to production requests and actions;
- **3D modeling using NX MCD and virtual model PLC implementation based on an inverse kinematics analytical approach**. In robotic manufacturing, model kinematic equations must be solved at a high rate for PLC execution; therefore, a closed-form solution is preferred over a numerical one;
- **SCADA DT design and functionality**. SCADA enhances DT by enabling monitoring, visualization, and Cloud Storage. It serves as a DT operator panel for simulation, data analysis, and remote operation. Additionally, it diagnoses and prevents problems, alerts operators, and improves A/DRC system reliability while reducing production costs and safety risks.
- **A/DRC Virtual Commissioning platform implementation**. Developing and simulating a DT model for VC enhances engineering, optimizes design process, and enables smarter testing of physical systems. Combined with HIL, IoT, and Cloud solutions, DT improves manufacturing by enabling precise control, remote monitoring, and prediction of physical processes;
- **Configuration and integration of protocols for data synchronization and validation**. Reliable DT communication is crucial for performance and accuracy. To address these challenges, clear protocols such as PROFINET IO, Ethernet, and WAN-Ethernet with OPC-UA are implemented to ensure stable, secure communication between local and Cloud systems, enabling efficient remote control and monitoring;
- **IoT-Cloud-based Control and data acquisition**. The IoT-Cloud platform enables real-time monitoring, remote access, data analytics, and cloud storage for A/DRC. Additionally, Cloud and Edge computing should be used to process data closer to the source, reducing latency, enhancing decision-making, and ensuring data security. Implementing these strategies improves DT applications and data quality, enhancing reliability, and automation.

This research work aims to evaluate whether intelligent digital technologies integrated into manufacturing applications, especially in Industrial robotics systems, like Virtual Commissioning, should be included in their suite of tools for testing the programs under various scenarios. Additionally, integrating a digital model with Cloud storage solutions can easily accommodate growing data requirements, enabling real-time data collection and monitoring of robotic cells equipped with IoT sensors. This facilitates faster decision making, quicker troubleshooting of potential issues, and a significant reduction in downtime associated with maintenance tasks. This includes optimizing operational tasks, addressing delays, enabling predictive maintenance, and reducing costs related to on-site commissioning.

The research presented is still ongoing, with the main objective of adapting the A/DRC assisted by ABB 120 IRM technology, currently implemented on a laboratory system, to align with the rapidly evolving developments in Industry 4.0 and 5.0. Through this study, we extended the degree of automation and efficiency by introducing smart manufacturing technologies, such as IoT and digitalization, into the manufacturing process. As a result, this approach leads to improved accuracy, predictability, robustness, and overall performance.

Although robotic cell Digital Twin technology with the HIL approach has been used and tested at the level of a laboratory, it can be extended to ensure its applicability to real-world industrial systems and various applications, ultimately enhancing operational efficiency and boosting productivity. The hardware and software configuration for the robotic cell Digital Twin approach with an HIL structure enhances robustness against defects and uncertainties across all subsystems, ensuring adaptability and flexibility-key characteristics of Industries 4.0 and 5.0.

## Figures and Tables

**Table 1:** Angle limits and velocities for ABB 120 IRM axes.

ABB 120 IRM Axes	Range	Max Speed (T)
**Axis 1 Rotation**	+165° to −165°	250°/s
**Axis 2 Arm**	+110° to −110°	250°/s
**Axis 3 Arm**	+70° to −110°	250°/s
**Axis 4 Wrist**	+160° to −160°	320°/s (420°/s)
**Axis 5 Bend**	+120° to −120°	320°/s (590°/s)
**Axis 6 Turn**	+400° to −400°	420°/s (600°/s)

**Table 2:** ABB 120 IRM D-H parameterization used to determine the homogeneous transformation matrix for each link (also known as the Denavit–Hartenberg matrix).

Joint	***α***_i_ [°]	***a***_i_ [**mm**]	***θ***_i_ [°]	***d***_i_ [**mm**]
***T_1_***(*θ*_1_)	−**π**/2	0	***θ***_1_	***d***_1_ = 290
***T_2_***(*θ*_2_)	0	**a**_1_ = 270	***θ***_2_ − **π**/2	0
***T_3_***(*θ*_3_)	−**π**/2	**a**_1_ = 70	***θ***_3_	0
***T_4_***(*θ*_4_)	**π**/2	0	***θ***_4_	***d***_4_ = 302
***T_5_***(*θ*_5_)	−**π**/2	0	***θ***_5_	0
***T_6_***(*θ*_6_)	0	0	***θ***_6_ + **π**	***d***_6_ = 72

## Data Availability

Data are contained within the article.
